# Integrating cellular and soluble immune signatures of major depression with and without recent suicide attempts

**DOI:** 10.1038/s41398-025-03601-2

**Published:** 2025-10-06

**Authors:** Aiste Lengvenyte, Marine Blaquiere, Jonathan Dubois, Marine Bonnin, Julie Bourret, Hind Hamzeh-Cognasse, Emilie Olié, Fabrice Cognasse, Nicola Marchi, Philippe Courtet

**Affiliations:** 1 Department of Emergency Psychiatry and Acute Care, Lapeyronie Hospital, CHU Montpellier, Montpellier, France; 2 Institute of Functional Genomics, University of Montpellier, CNRS, INSERM, Montpellier, France; 3 Psychiatric Clinic, Institute of Clinical Medicine, Faculty of Medicine, Vilnius University, Vilnius, Lithuania; 4 Université Jean Monnet, Mines Saint-Étienne, INSERM, Saint-Étienne, France; 5 Etablissement Français du Sang Auvergne-Rhône-Alpes, Saint-Étienne, France; 6 Fondation Fondamental, Créteil, France

**Keywords:** Diagnostic markers, Depression

## Abstract

Suicide attempts (SA) during major depressive episodes (MDE) pose a significant clinical challenge, yet its underlying biological mechanisms, particularly those associated with recent SA, remain poorly understood. In this case-control study (n = 106), we compared the immunological profiles associated with MDE without SA (n = 36), MDE with a recent SA (n = 35), and a healthy controls (n = 35). Fresh blood samples were analyzed for total cell count and using flow cytometry (FACS) to assess specific cell surface markers. Plasma proteins were quantified using multiplex ELISA or Quanterix. Multiple factorial analysis (MFA) identified three primary dimensions structuring the biological markers: soluble immune and growth factors, cellular immunity (cell ratios, CD45^+^, CD3^+^, CD8^+^, CD14^+^), and neuroinflammatory markers (GFAP, NfL, CD4+). These dimensions differentiated MDE patients from healthy controls regardless of SA status. Three parallel feature selection analyses were used to examine whether individual biomarkers could differentiate between the subgroups. Lower percentages of CD3^+^, CD8^+^CD3^+^, higher basophil count, and lower serotonin levels differentiated MDE without SA from healthy controls. An increased neutrophil-to-lymphocyte ratio and basophil count, and lower serotonin levels differentiated recent SA from healthy controls. We found no significant differences between MDE patients with and without SA, though exploratory analyses identified individual markers, such as platelet-derived factors, that warrant further investigation. These findings provide new insights into the immune landscape of MDE and recent SA and underscore the need for future longitudinal studies to disentangle state- and trait-like biological changes associated with SA.

## Introduction

Major depressive episodes (MDE) are prevalent, disabling, and linked to premature mortality due to both suicide and increased vulnerability to medical disorders [1, 2]. A significant proportion of patients experience suicidal crises, leading to suicide attempts (SA) and, in some cases, death. The pathophysiology of MDE and SA is multifactorial, involving complex and interrelated mechanisms.

MDE is associated with alterations in peripheral immune-inflammatory markers and growth factors, with the most robust evidence showing increased C-reactive protein (CRP), interleukin-6 (IL-6), and tumor necrosis factor-α (TNF-α) [3–5]. Positron emission tomography and post-mortem studies reveal increased neuroinflammation in MDE and individuals who died by suicide [6–9], suggesting a peripheral-brain immunological interplay, possibly facilitated by increased vascular permeability in MDE [10–12]. Inflammation and cerebrovascular permeability are emerging contributors to brain disorders [11, 13, 14].

MDE patients with SA history show elevated blood levels of inflammatory markers such as CRP, IL-6, and the neutrophil-to-lymphocyte ratio (NLR) compared to those without SA [4, 15], with some novel markers also emerging [16, 17]. However, integrated analyses of cellular and soluble immune components are rare, limiting a comprehensive understanding of immune-inflammatory pathways in MDE and SA.

The period shortly after an SA represents both a critical intervention window and an opportunity to elucidate the biological underpinnings of SA and its distinction from non-suicidal MDE [18]. Despite its importance, studies investigating immune changes during this acute phase are scarce. Our previous work showed that past-month SA was linked to specific immune alterations, not found in individuals with more temporally distal SA [16]. This supports the notion that markers temporally linked to SA may better reflect acute risk than trait-like markers. While our earlier study found no CRP differences between individuals with recent vs. earlier SA [19], other evidence suggests that past-week SA is associated with cognitive alterations absent in earlier SA [20].

Peripheral inflammation has been implicated in brain-blood barrier dysfunction and neural connectivity alterations, which may increase SA risk [21, 22]. Stress-induced immune activation and its links to suicidal behaviors further highlight the role of inflammation in acute suicidal states [23, 24]. However, the extent to which immune-inflammatory alterations distinguish recent SA from non-suicidal MDE remains unclear. We hypothesized that MDE patients with recent SA would exhibit a distinct peripheral immune-inflammatory profile compared to MDE patients without SA and healthy controls. Specifically, we expected that recent SA would be marked by increased immune activation and neuroinflammation, reflecting state-like alterations associated with suicidal crisis. By delineating biological pathways in MDE with and without SA, this study aimed to refine our understanding of suicidal behaviors and contribute to biomarker-based risk assessment and intervention strategies.

## Materials and methods

### Study design and participants

This monocentric, cross-sectional, case-control study recruited participants from the Department of Emergency Psychiatry and Acute Care, Montpellier University Hospital, France, between November 2019 and July 2022. Participants were matched by sex and age into three groups: (1) individuals with a current MDE, determined by the Structured Clinical Interview for DSM-5 (SCID-5) [25], and a first SA within the past eight days, determined by the Columbia Suicide Severity Rating Scale (C-SSRS) [26]; (2) individuals with a current MDE without a lifetime SA; and (3) healthy volunteers with no psychiatric history (SCID-5) or SA (C-SSRS) [26]. MDE patients with major depressive disorder and bipolar disorder were included due to the transdiagnostic nature of SA and and potentially shared biological markers for MDE and SA among diagnoses [15, 16, 27, 28]. Inclusion criteria were age 18–55 years, social security affiliation, and informed consent. Exclusion criteria included chronic inflammatory/autoimmune diseases, active infections, immune-modulating treatments, severe alcohol or substance use disorder, schizophrenia/schizoaffective disorder, and pregnancy/lactation. Patients were medically stabilized before psychiatric assessment, and those with significant traumatic brain injury or life-threatening drug intoxication requiring intensive care were ineligible. Suicidal ideation was not an exclusion criterion for the MDE group, and its prevalence was recorded for all groups.

MDE patients with recent SA were included based on an 8-day post-attempt timeframe, balancing biological relevance and practical constraints. Since recruitment was first-come in clinical settings, and healthy volunteers participated voluntarily, precise records of non-included individuals were unavailable. However, all eligible patients within this timeframe who met inclusion/exclusion criteria were invited. MDE patients without SA were recruited during hospitalization or outpatient consultation. Healthy volunteers were recruited via online/hospital advertisements. The study was approved by CHU Montpellier’s Institutional Review Board and registered (NCT04137458). Participants provided written consent and received a 45-euro compensation. Findings are reported according to the Strengthening the Reporting of Observational Studies in Epidemiology guidelines [29].

### Sample size

As an exploratory study, sample size estimation was based on a detectable fold change of 1.5 for pairwise group comparisons, 80% power, 0.05 significance level, and anticipated biological and technical variability (≤75%), requiring 35 individuals per group [30].

### Assessments

A trained mental health professional conducted evaluations. Psychiatric diagnoses were determined by SCID-5 [25], depression severity by BDI [31], and suicidal ideation by BDI item #9 (threshold: ≥2). SA history was assessed using the C-SSRS [26], with recent SA defined as occurring within eight days before evaluation. Additional data included sociodemographics, treatment, somatic comorbidities, BMI, tobacco use, and abdominal perimeter.

Fasting venous blood samples (8:00–10:00 AM) were collected and processed per hospital standards. Complete blood counts and CRP were analyzed immediately (C800 Roche platform), including absolute eosinophils, basophils, neutrophils, and platelets. Based on prior associations with MDE and SA, we calculated NLR, platelet-to-lymphocyte ratio (PLR), and monocyte-to-lymphocyte ratio (MLR) [15].

### Flow cytometry

We performed flow cytometry on fresh blood within 3 h of collection, with each participant’s sample processed and analyzed individually on the day of blood draw. A 500 µl whole blood sample from an EDTA tube was mixed with 100 µl FcR Blocking buffer (130-059-901, Miltenyi Biotec, Germany) for 15 s, followed by incubation with fluorochrome-conjugated antibodies for CD45+ (VioBlue, CD3-PE, CD14-APC) and CD3+ (VioBlue, CD4-PE, CD8-APC) (Miltenyi Biotec, Germany) for 15 min. After incubation, 100 µl of CAL-LYSE Lysing solution (GAS010 Life Technologies, USA) was added, and samples were incubated for 10 min before adding 1 mL of deionized sterile water and storing at 4 °C for up to 4 h until flow cytometry readout. Flow cytometry was performed using a MACSQuant cytometer (Miltenyi Biotec, Germany) at the Montpellier Ressources Imagerie (MRI) platform. The instrument operates with laser excitation at 405 nm (Vioblue detection: 450/50 nm), 488 nm (PE detection: 585/40 nm), and 640 nm (APC detection: 655–730 nm). The MACSQuant system is a high-performance flow cytometer with automated fluorescence detection, compensation, and calibration protocols, ensuring rigorous quality control. To minimize technical variability, we performed daily instrument calibration using fluorescence control beads, applied session-specific compensation controls to correct spectral overlap, and adhered to strict standardization of sample processing and staining protocols. Immune cell populations were normalized as percentages relative to total CD45+ leukocytes or relevant parent subsets to ensure comparability. Each sample was analyzed in triplicate, with at least 30,000 events collected, and gating on FSC-A/FSC-W was applied to exclude doublets and debris. The following immune cell populations were analyzed: peripheral blood mononuclear cells (CD45+), T-lymphocytes (CD3+), T-helpers (CD4+), T-killers (CD8+), and monocytes (CD14+). Data were processed using Flowing Software (Turku Bioscience Centre, University of Turku, Finland). Personnel performing flow cytometry were blind to study groups.

### Plasma and serum protein analyses

We used 50 µl of serum samples (from EDTA tubes) to measure Glial Fibrillary Acidic Protein (GFAP) and Neurofilament light (NfL) protein concentrations. These analyses were conducted by the Clinical Proteomics Platform (PPC, Montpellier, France) using the Simoa Neurology 2-Plex Kit (103520, Quanterix, Massachusetts, USA) on the HD-X Analyzer system. In parallel, plasma samples were centrifuged at 3000 x g at room temperature for 20 min and stored at −80 °C for batched analysis using ELISA assay technology. Based on existing literature and our previous findings, we measured the following plasma markers, which have been implicated in depression and SA pathophysiology [3, 4, 16, 17, 32]: interferon-$\gamma$ (IFN- $\gamma$), TNF-$\alpha$ IL-1$\beta$, IL-2, IL-4, IL-6, monocyte chemoattractant protein-1 (MCP-1), transforming growth factor-1$\beta$ (TGF-1$\beta$), platelet-derived growth factor-AB (PDGF-AB), PDGF-BB, thrombospondin-1 (TSP-1), thombospondin-2 (TSP-2), RANTES (regulated on activation, normal T cell expressed and secreted, also called CCL5), uteroglobin, annexin, serotonin, and centrin-2. All parameters were assayed in duplicate wells. Merch Millipore (Luminex), Bio-Techne SA (Chatillon sur Seiche, France), and Tecan France (Lyon, France) (ELISA) platforms were used. Details of the platforms and kits used for plasma protein analysis are provided in the Supplemental Table S1. The inter-plate coefficient of variation (CV) was calculated, with low levels of inter-plate variability for all analytes (CV < 2). Personnel performing protein analyses were blind to study groups.

### Statistical analysis

Statistical analyses were performed using R statistical computing software, version 4.3.0 (R foundation for Statistical Computing, Vienna, Austria). Outliers were evaluated using summary statistics, and skewed variables were log-transformed to improve normality and mitigate the influence of extreme values. Group variance was comparable after transformation. Given that chosen statistical methods, including Multiple Factor Analysis (MFA), stepwise selection using the Akaike Information Criterion (stepAIC; forward/backward), regularized Least Absolute Shrinkage and Selection Operator (LASSO) selection, and Bayesian Stochastic Search Variable Selection (SSVS) are robust to extreme values, no further exclusion or winsorization was applied. Protein marker values below the level of quantification were treated based on the extent of undetectability. If <20% of values were non-detectable, single imputation was performed using a censored log-normal distribution to preserve data continuity [33]. If ≥20% of values were non-detectable, categorical variables (2–3 balanced groups) were created to avoid unreliable extrapolations. There were no missing values in protein analyses. Missing values in flow cytometry data (n = 7), and complete blood count (n = 3) resulted from technical issues, which occurred independently of clinical characteristics. To ensure an unbiased approach to handling missing data, we applied MFA for imputation, as the probability of missingness was unlikely to be systematically related to the biological variables of interest. Numbers of undetectable and missing values, as well as the imputation method are presented in Supplemental Table 2. We directly compared statistical significance and group means between non-imputed and imputed datasets, which showed consistent results (Supplemental Tables S3–S5). We prioritized imputation over complete case analysis to preserve statistical power, maintain cohort representativity, and minimize potential selection bias. Descriptive analysis of the three groups included means with standard deviations (s.d.) and counts (%). As an exploratory step, the three groups were compared using univariate t-tests or Pearson’s Chi^2^ tests.

To address high dimensionality and correlation, MFA was applied to identify biologically meaningful, uncorrelated dimensions. MFA is a principal component method that balances differences in the number of active variables per domain by forming active groups. Meaningful fators were selected using Kaiser’s criterion (eigenvalue > 1) and characterized via correlation ranking and F-tests. Group differences were also tested on each component using one-way ANOVAs. Adjusted logistic regressions were then fitted for all group pair comparisons to test for differences between groups in the extracted meaningful MFA components. Chi^2^ tests, Odds Ratios (OR), and 95% Confidence Intervals (95% CI) are reported.

To determine whether specific biological markers were associated with each study group, we contrasted the results of three variable selection methods that were performed in parallel to prevent overfitting and false positive associations: stepAIC, LASSO, and SSVS [34]. Sex and age were forced into models, and for MDE with recent SA vs. MDE without SA, we additionally adjusted for depression severity. Markers identified by all three methods were considered significant. P-values were two-sided, with p < 0.05 considered statistically significant. Bonferroni correction was applied to multivariate models.

## Results

### Sample characteristics

The analysis included 106 participants: 36 individuals with MDE with recent SA, 35 individuals with MDE without a history of SA, and 35 healthy volunteers. Among MDE patients with recent SA, three had made a lethal SA (e.g., phlebotomy), and seven required medical stabilization in intensive care prior to inclusion. Due to the small number of cases with severe or lethal attempts, no subgroup analyses by SA severity or lethality were conducted. Instead, all participants with recent SA were analyzed together to identify shared biological alterations associated with this acute phase. The three groups did not differ significantly regarding sex, mean age, abdominal perimeter, BMI, or smoking status. As expected, both groups with MDE exhibited higher levels of depression and suicidal ideation compared to healthy volunteers, and most of them were on pharmacological treatment, while healthy controls were medication-free. MDE patients with recent SA were more likely to receive anxiolytic/hypnotic treatment than patients without SA history, with a trend towards greater depression severity. Sample characteristics are summarized in Table 1.

**Table 1:** Sample characteristics.

Participant Characteristics	Healthy controls	Major depressive episode without suicide attempt	Major depressive episode with suicide attempt	p-value
Number of participants	35	35	36	..
Female sex, *n (%)*	26 (74.3)	26 (74.3)	27 (75)	1
Age, years, *mean (s.d.)*	34.114 (10.701)	33.706 (10.842)	32.167 (12.852)	0.74
BMI, kg/m^2^,l *mean (s.d.)*	22.88 (5.82)	23.49 (7.70)	23.82 (7.47)	0.42
Waist circumference, cm, *mean (s.d.)*	86.5 (10.871)	93.5 (15.951)	85.714 (14.072)	0.14
Metabolic syndrome, *n (%)*	1 (2.9)	3 (8.6)	3 (8.3)	0.59
Tobacco user, current, *n (%)*	8 (22.9)	15 (42.9)	14 (38.9)	0.42
Family history of suicidal behaviour, *n (%)*	10 (28.6)	11 (31.4)	15 (41.7)	0.70
Current hospitalisation, *n (%)*	0 (0)	23 (65.7)	32 (88.9)	<0.001
Psychiatric diagnoses, *n (%)*	..	..	..	..
Major depressive disorder	0 (0)	29 (82.9)	31 (86.1)	<0.001
Bipolar disorder	0 (0)	6 (17.1)	5 (13.9)	<0.001
Anxiety disorder	0 (0)	24 (68.6)	20 (55.6)	<0.001
Alcohol or substance use disorder	0 (0)	6 (17.1)	9 (25.0)	<0.001
Eating disorder	0 (0)	6 (17.1)	8 (22.2)	<0.001
Psychiatric scales				
BDI total score, *mean (s.d.)*	0.829 (1.317)	17.429 (7.942)	20.556 (6.097)	<0.001
Suicidal ideation (BDI item #9), *n (%)*	0 (0)	5 (14.3)	8 (22.2)	<0.001
Childhood trauma questionnaire, moderate of severe, *n (%)*	..	..	..	..
Emotional abuse	3 (8.6)	16 (45.7)	29 (80.6)	<0.001
Physical abuse	2 (5.7)	10 (28.6)	16 (44.4)	0.01
Sexual abuse	2 (5.7)	10 (28.6)	12 (33.3)	0.05
Emotional neglect	0 (0)	11 (31.4)	18 (50.0)	<00.1
Physical neglect	7 (20)	23 (65.7)	33 (91.7)	0.004
Current treatment, *n (%)*	..	..	..	..
Anxiolytics and hypnotics	0 (0)	22 (62.9)	32 (88.9)	<0.001
Antidepressants	0 (0)	29 (82.9)	26 (72.2)	<0.001
Mood stabilizers	0 (0)	14 (40)	22 (61.1)	<0.001

Mean (s.d.) and N (%) are presented for quantitative and qualitative variables.

In unadjusted pairwise comparisons, MDE patients without SA history exhibited lower plasma levels of IFN-$\gamma$, IL-1$\beta$, IL-2, PDGF-AB, RANTES, PDGF-BB, serotonin, TGF- $\beta$1, TSP-1, alongside higher serum GFAP levels, compared to healthy volunteers. These patients also showed alterations in blood cell counts, including higher basophil counts, a lower percentage of CD3^+^ among single cells, higher CD4^+^ and lower CD8^+^ percentages among CD3^+^ cells, and an elevated CD4^+^/CD8^+^ ratio (Supplemental Table S3). Similarly, patients with recent SA demonstrated lower levels of PDGF-AB, RANTES, PDGF-BB, serotonin, TGF-$\beta$1, and TSP-1 compared to healthy controls. They also had a lower CD3^+^ percentage among single cells and a lower CD8^+^ percentage among CD3^+^cells, alongside higher counts of total nucleated cells, neutrophils, and basophils, as well as increased NLR (Supplemental Table S4). Comparing MDE patients with recent SA to those without SA history, the former had higher TNF-α and PDGF-AB levels and an increased NLR in the least strict approach (Supplemental Table S5). However, after applying a conservative Bonferroni correction (p < 0.001), only the lower serotonin levels in the clinical groups versus healthy volunteers remained statistically significant.

### Multiple factorial analysis

**Fig. 1:**
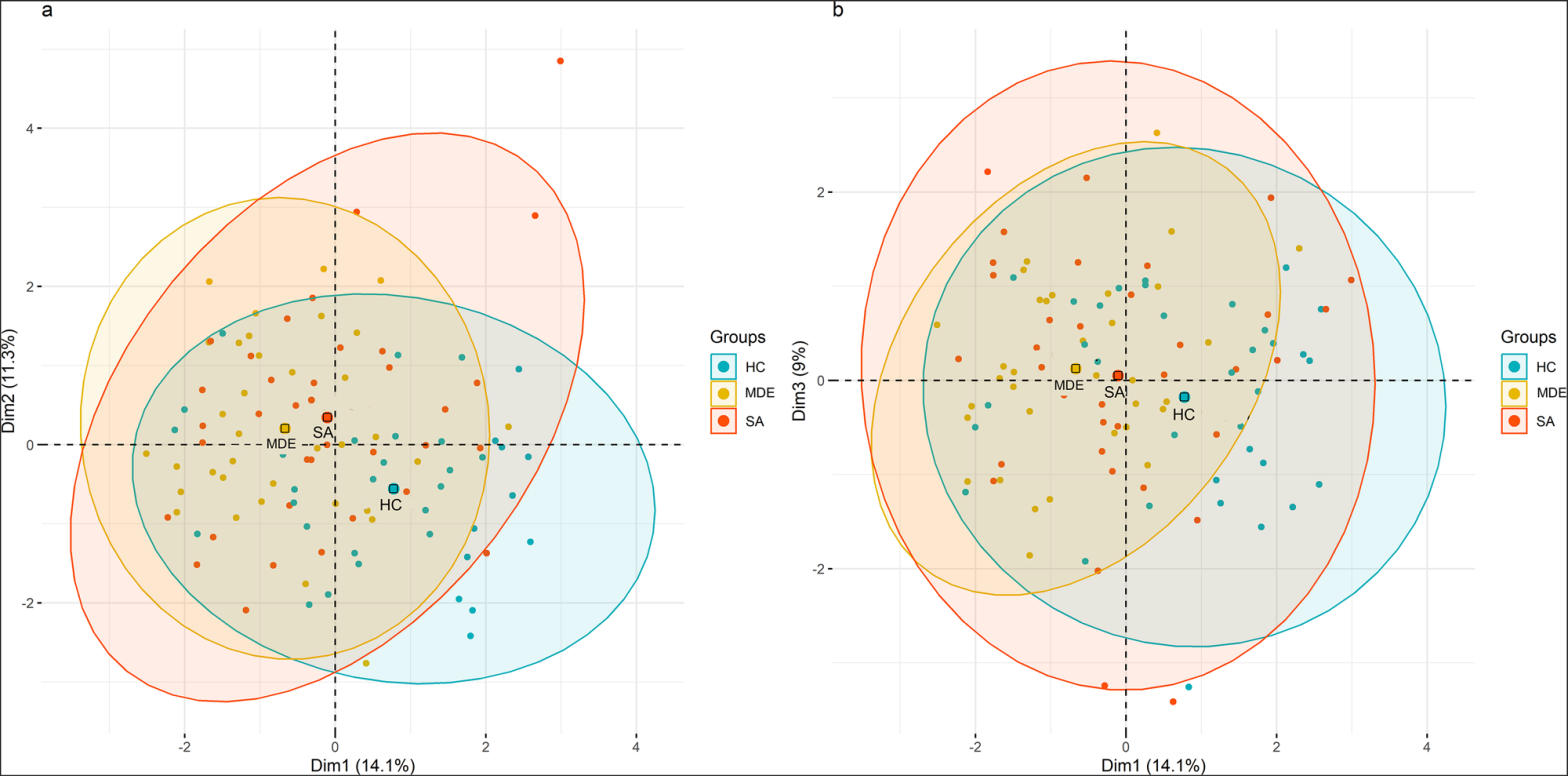
Representation of individuals across the first three Multiple Factor Analysis dimensions by study group. **a** Projection of individuals onto dimensions 1 and 2. Each point represents an individual, with colors corresponding to study groups (healthy controls, HC; major depressive episode without suicide attempt history, MDE; major depressive episode with recent suicide attempt, SA). Ellipses indicate 95% confidence intervals for group distributions. **b** Projection of individuals onto dimensions 2 and 3. The first three Multiple Factor Analysis dimensions capture the primary sources of variance across the dataset, allowing visualization of group separation.

We conducted Multiple Factor Analysis (MFA) to identify dimensions structuring the sets of variables examined. According to the Kaiser eigenvalue criterion, three major components were retained, explaining 34.4% of the total variance (14.1, 11.4, and 9.0% for the first, second, and third dimensions, respectively). MDE patients without SA history (OR, 0.206; 95% CI, 0.08 to 0.433, p < 0.0001) and recent suicide attempters (OR, 0.303; 95% 269 CI, 0.14 to 0.585, p = 0.0002) were distinctly separated from healthy controls on the first two dimensions. On the second dimension, recent suicide attempters were slightly better discriminated from healthy controls (OR, 3.305; 95% CI, 1.693 to 7.716, p = 0.0002) than MDE patients without SA history (OR, 2.689; 95% CI, 1.333 to 6.249, p = 0.005). On the third dimension, both MDE patients without SA history and suicide attempters had higher scores compared to healthy controls. However, only the difference between MDE patients without SA history and healthy controls reached statistical significance (OR, 2.519; 95% CI, 1.225 to 6.055, p = 0.01), while the difference between recent suicide attempters and healthy controls did not (OR, 1.644; 95% CI, 0.881 to 3.286, p = 0.12). Detailed findings for the three dimensions concerning the study groups are presented in Fig. 1 and Supplemental Table S6.

**Fig. 2:**
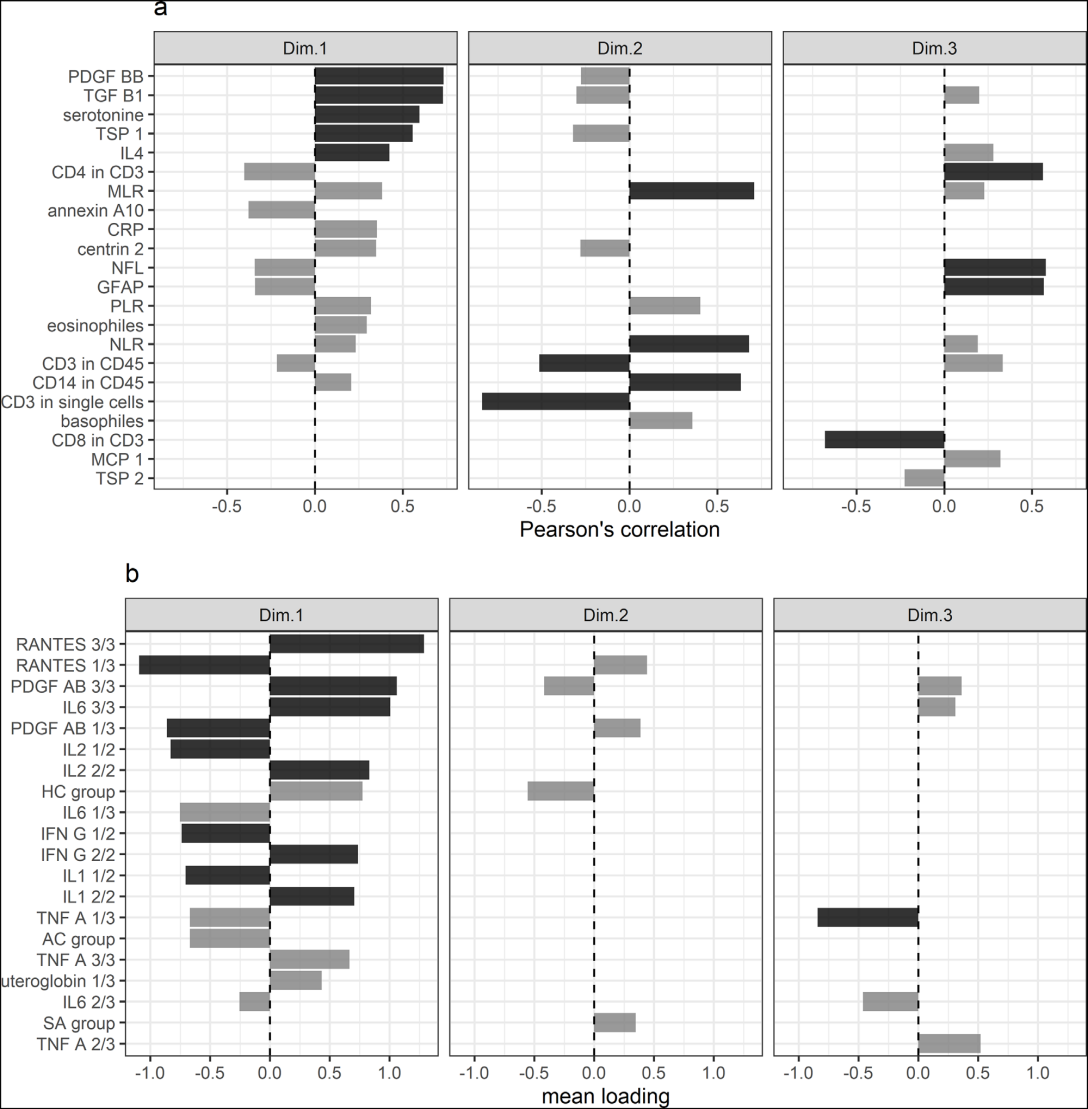
Characterization of the three meaningful Multiple Factor Analysis dimensions. **a** Correlations between each Multiple Factor Analysis dimension and quantitative variables, highlighting key contributors to the variance structure. **b** Mean loading of categorical variables within each dimension. The fraction after qualitative variable names indicates their ordinal level relative to the total number of levels. Statistically significant associations (p < 0.001, Bonferroni-corrected) are marked with black bars, while gray bars represent variables that were informative but did not reach statistical significance.

Age- and sex-adjusted analyses revealed that the first dimension primarily captured variations in plasma immune modulation and growth factors, with the strongest contributions from elevated levels of PDGF-BB, TGF-$\beta$1, TSP-1, IL-4, serotonin (Fig. 2a), RANTES, PDGF-AB, IL-6, IL-2, and IFN-$\gamma$ (Fig. 2b). The second dimension was characterized by a shift in blood immune cell composition, favoring innate over adaptive immunity, as indicated by higher MLR, NLR, basophil counts, CD14^+^ percentages among CD45^+^ cells, and lower CD3^+^ percentages among CD45^+^ and single cells (Fig. 2a). The third dimension was associated with increased neuroinflammation, reflected by elevated serum levels of NfL, GFAP, and a higher proportion of CD4^+^ among CD3^+^ cells (Fig. 2a).

### Feature selection analyses

**Fig. 3:**
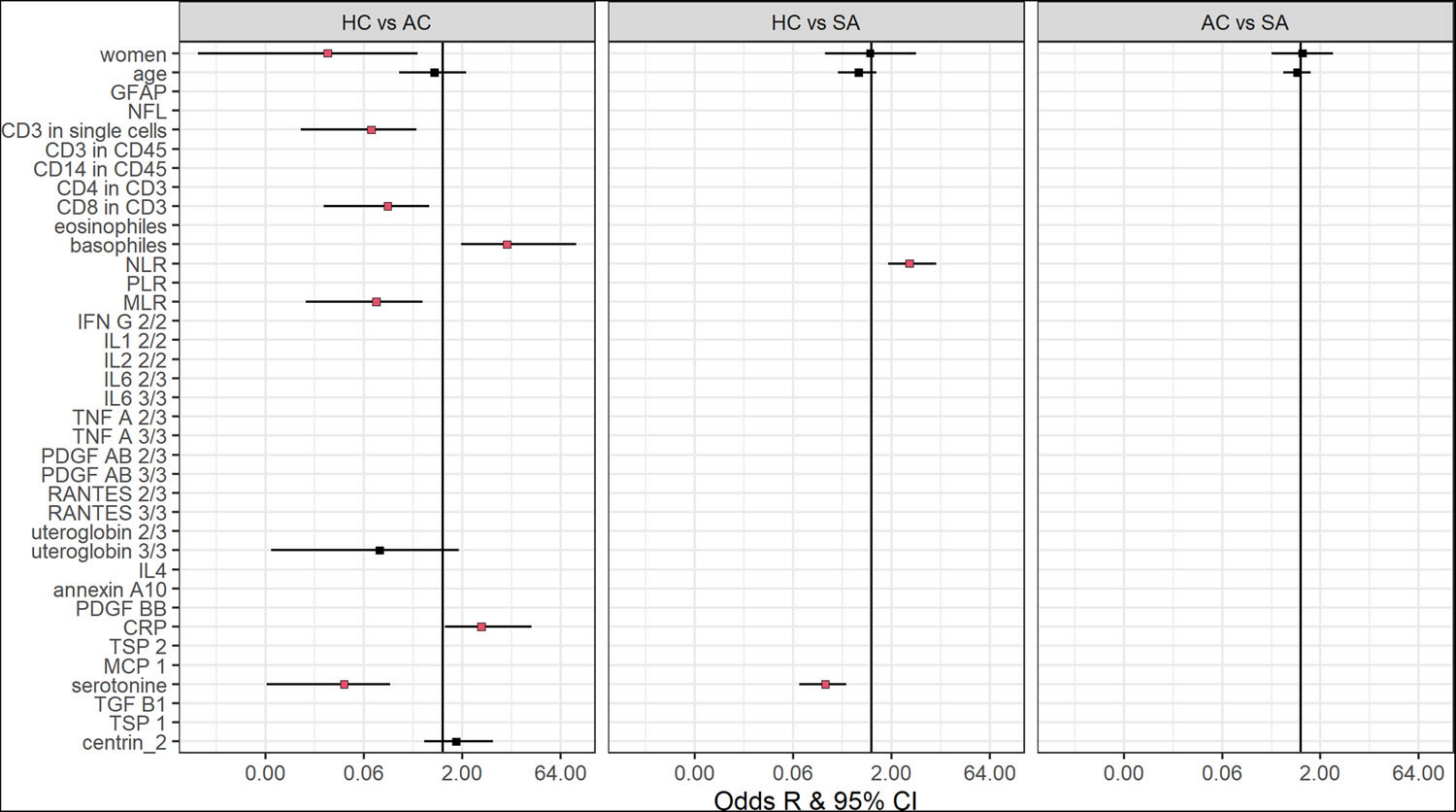
Odds ratios and 95% confidence intervals from multivariate models following the SSVS selection procedure. Displayed are the odds ratios (ORs) and 95% confidence intervals (CIs) for the three group comparisons (healthy controls HC, major depressive episode without suicide attempt history MDE, major depressive episode with recent suicide attempt SA).), derived from Bayesian Stochastic Search Variable Selection (SSVS). All models were adjusted for sex and age. Additionally, depression severity was included as a covariate when comparing MDE versus SA groups.

In parallel to MFA, we conducted feature selection analyses to identify individual markers characteristic of each study group. Three selection methods were applied - stepAIC selection, LASSO, and SSVS – each adjusted for age and sex. Results from the SSVS method are shown in Fig. 3, and comparisons from all three methods for each pairwise group contrast are presented in Supplemental Figs. S1, S2, and S3. Only markers selected by all three methods were considered significant. Higher NLR and basophil count differentiated MDE patients with recent SA from healthy controls. In contrast, lower percentages of CD3^+^, CD8^+^ in CD3^+^, higher CRP levels, and higher basophil counts distinguished MDE patients without SA history from healthy volunteers. No markers were found to differentiate MDE patients with recent SA from MDE patients without SA history.

### Sensitivity analysis in patients with major depressive disorder

We conducted a sensitivity analysis excluding individuals with bipolar disorder. The overall MFA structure remained unchanged, with the first three dimensions explaining 34.5% of the variance. The same variables drove each dimension (Supplemental Fig. S4). The positioning of MDE patients with SA history, MDE patients without SA history, and healthy controls on these dimensions remained consistent with the main analysis (Supplemental Fig. S5). In multivariate models examining associations between study groups and MFA dimensions, the associations remained consistent with the main analysis, except for the difference between healthy controls and MDE patients without SA history, which showed the same tendency but lost statistical significance (Supplemental Table S7). In feature selection, the same group differences between MDE patients and healthy controls were retained (Supplemental Fig. S6).

## Discussion

This study examined a broad range of immune modulation biomarkers to identify peripheral signatures of MDE with and without recent SA. Using a multifaceted approach, we identified three main dimensions: immune modulation and growth factors, cellular immunity, and neuroinflammation. MDE was characterized by reductions in immune modulation proteins and growth factors, a shift toward innate immunity, and subtle neuroinflammation. No significant dimensional differences emerged between MDE patients with and without SA history, though several markers distinguished clinical groups from healthy controls.

### Immune modulation and growth factors

MDE patients with and without SA history scored lower than healthy controls on the immune modulation and growth factor dimension. Key markers associated with this dimension were also lower in clinical groups in the univariate pairwise comparisons, but only lower serotonin levels emerged as a significant differentiator in the variable selection approach. This aligns with prior research linking serotonergic dysfunction to MDE and SA [16, 35], though medication effects in patient groups may confound this finding [36].

Our findings on lower scores in the immune modulation and growth factors dimension in MDE are consistent with previous reports of reduced plasma TGF-β1 levels in depression [37–39]. TGF-β1, which regulates neurogenesis and synaptogenesis, is linked to cognitive decline when reduced [40]. Conversely, stress-resilient mice show higher TGF-β1 levels [41, 42]. Similarly, lower IL-4 levels, another key marker in this dimension, are commonly seen in MDE [5].

Markers associated with this dimension—TGF-β1, TSP-1, PDGF-BB, and RANTES—are secreted by activated platelets [43, 44]. While a meta-analysis found no significant differences in RANTES levels between depressed patients and controls [3], some studies have linked lower RANTES levels with suicidal ideation in MDE [16, 45]. Research on other markers in MDE is limited. For instance, PDGF-AB and TSP-1 levels are highly correlated, yet studies report varying results, with one finding reduced TSP-1 in MDE and another noting increased PDGF-AB levels [16, 46, 47]. Similarly, efforts to differentiate major depressive disorder from bipolar disorder using PDGF-BB and TSP-1 have yielded inconsistent results [48, 49]. Notably, antidepressant treatment may increase PDGF-BB [50], suggesting that the lower scores observed in our study are not merely treatment effects.

Interestingly, CRP, a widely recognized marker in MDE [51], was retained in feature selection but did not strongly associate with any dimension, suggesting an independent role.

### Cellular shift towards innate immunity in depression

Both MDE groups showed a shift towards innate immune dominance, with higher scores than healthy controls on the second dimension. This aligns with prior findings of increased neutrophils and monocytes alongside reduced CD3+ and CD8+T-cell percentages in depression [52–55]. Functionally, this shift may indicate a system primed for immediate stress response but with reduced adaptive immunity, impairing long-term immune regulation. Additionally, lower CD3+ and CD8+ cell percentages and higher basophil counts distinguished MDE patients without SA from healthy controls. Depression has been associated with innate immune overexpression and adaptive immune suppression, along with lymphocyte deficiencies and monocyte activation [52, 56, 57].

While our panel was limited in immune cell phenotyping, recent studies suggest that monocyte subsets (non-classical and intermediate), as well as T-cell exhaustion markers, play a role in MDE [28]. Further, suicidal ideation and SA have been linked to increased T-cell exhaustion, independent of depressive symptoms [58]. Though we did not measure these markers, they may help distinguish SA subtypes. These findings underscore the need for expanded immune phenotyping, particularly of monocyte subsets and T-cell function, to refine our understanding of immune dysregulation in MDE and SA.

### Neuroinflammation

MDE patients scored higher on the third dimension, driven by increased blood levels of NfL, GFAP, and CD4+ cells. Consistent with prior findings, elevated NfL and GFAP have been reported in mood disorders [59–63]. While not specific to MDE, these markers reflect neuroaxonal injury (NfL) and astroglial activation (GFAP), suggesting a link between MDE and neurodegenerative or neuroinflammatory processes [60, 64, 65]. Though less pronounced than in neurodegenerative diseases, the subtle increases observed here suggest cellular maladaptation in depression.

The correlation of CD4+ cells with this dimension highlights a potential link between peripheral immunity and neuroinflammation. Elevated CD4+ concentrations have been reported in MDE [52, 66] and associated with stress-related behaviors and increased infiltration into the CNS under chronic stress [12, 67]. Once in the brain, CD4+ cells can sustain inflammation and impair blood-brain barrier integrity, aligning with post-mortem findings of abnormal T-cell densities in mood disorder patients [68]. This neuroinflammation dimension was distinct from the innate immune shift and immune modulation/growth factor reduction, underscoring the complex interplay between immune dysregulation and neuroinflammation in MDE.

### Differences between patients with depression with recent suicide attempt history from those without

The lack of significant immune-inflammatory differences between MDE patients with and without recent SA should be interpreted cautiously, given the exploratory nature of this study and sample size limitations. Subtle differences may not have been detected due to limited statistical power. Additionally, unlike most studies, we recruited patients shortly after their first SA, capturing a post-crisis phase in which immune activity may shift toward homeostasis. Crisis resolution and increased social support during hospitalization may temporarily reduce stress-related immune alterations [69–72]. Furthermore, MDE patients with recent SA did not differ in current suicidal ideation from those without SA history. Suicidal ideation, which fluctuates acutely and is a key clinical component of suicidal crises, may interact with immune-inflammatory processes [15, 23, 45]. Although MDE patients with recent SA had a trend toward greater depression severity, this difference was not statistically significant. Given the overall high symptom burden in both groups, subtler clinical variations may have been masked. Additionally, SA is often linked to depression variability rather than severity alone, which may explain the lack of clear biological distinctions [73].

While psychiatric comorbidities were similar across MDE groups, patients with recent SA were more likely to receive anxiolytic/hypnotic treatment, potentially reflecting greater clinical instability. However, these medications have also been associated with increased SA risk [74], suggesting potential bidirectional effects. Although not statistically significant, patients with recent SA also had higher use of antipsychotics and mood stabilizers and lower antidepressant use, possibly reflecting factors like borderline personality disorder or treatment-resistant depression, for which these medications are often prescribed [75]. Since the vast majority of MDE patients were on pharmacological treatment, while healthy controls were medication-free, treatment effects cannot be excluded. Given the immunomodulatory properties of antidepressants and antipsychotics, treatment may have influenced inflammatory markers [36, 76, 77]. Due to the unsupervised nature of MFA and lack of treatment in the healthy control group, treatment effects could not be directly adjusted for, and differences between MDE and healthy controls may have been attenuated.

Although no significant dimensional differences emerged between MDE patients with and without SA, higher NLR levels consistently differentiated recent SA patients from healthy controls across all three variable selection approaches. This aligns with prior findings linking NLR to SA risk [15, 78]. NLR reflects systemic immune activation and has been associated with stress-induced inflammation and childhood trauma, a major risk factor for SA [79]. In addition, while no markers consistently distinguished MDE with and without SA history across all variable selection methods, exploratory results identified TNF-α, PDGF-AB, PDGF-BB, and TSP-1 as potential differentiators. Given their platelet-derived origins, PDGF-BB, PDGF-AB, and TSP-1 may suggest a mechanistic link between platelet activation, vascular homeostasis, and SA [16]. These markers have been implicated in blood-brain barrier permeability and neurovascular inflammation, which could contribute to neuronal dysfunction and mood dysregulation during suicidal crises [10, 80]. Conversely, TNF-α, a pro-inflammatory cytokine, signals systemic immune activation. While preliminary, these findings highlight potential pathways that warrant further investigation into their role in SA pathophysiology.

### Limitations

Several limitations should be considered when interpreting these findings. First, while clinical groups were medicated, healthy controls were not, making it difficult to exclude medication effects. Second, the relatively small sample size limits statistical power. To mitigate this, we applied dimensionality reduction (MFA) and three complementary variable selection methods. However, larger cohorts are needed to validate findings, enable diagnosis-based stratification, and detect small effect sizes that may explain the lack of robust differences between MDE patients with and without SA history. Third, we did not adjust for suicidal ideation, which fluctuates and may influence immune-inflammatory markers [15, 23, 45]. Future studies should consider stratifying patients not only by SA history but also by suicidal ideation severity and variability to refine its role in biomarker profiles. Fourth, while individuals with severe SA-related complications were excluded, milder medical consequences were not systematically recorded, limiting our ability to determine their biological impact. Additionally, borderline personality disorder, a key SA risk factor, and longitudinal illness characteristics (e.g., recurrence, treatment response) were not assessed. Although measured psychiatric comorbidities were comparable between MDE groups groups, differences in medication use, particularly the higher prescription rates of anxiolytics/hypnotics, antipsychotics, and mood stabilizers in the SA group, may reflect crisis-driven treatment or underlying clinical heterogeneity. These medications, which have immunomodulatory effects, could influence both SA risk and immune-inflammatory findings.

Additionally, some biomarkers had high proportions of undetectable values and were analyzed as categorical variables, potentially reducing statistical power. While strict standardization minimized flow cytometry variability, inter-day differences cannot be fully excluded as samples were processed individually rather than in batches. Our flow cytometry panel was also limited to major immune populations, preventing analysis of monocyte subsets, regulatory T cells, T helper subtypes, NK cells, and memory T-cell compartments. More comprehensive immune profiling could uncover subset-specific alterations not captured here. Given the emerging role of monocyte subset differences and T-cell exhaustion markers in MDE and SA [28, 58], higher-resolution immune phenotyping could provide critical insights.

Finally, the cross-sectional design prevents causal inferences and limits insight into temporal immune-inflammatory changes before and after SA. While capturing the acute post-SA phase offers a unique perspective, this period may primarily reflect transient responses rather than stable biological alterations. Longitudinal studies with repeated assessments across acute and post-crisis phases are needed to better characterize these dynamics. Lastly, the observed dimensions may encompass distinct psychiatric symptom subdomains, and their construct validity warrants further investigation. Future work should refine these dimensions in larger, symptom-stratified cohorts to clarify their biological relevance.

### Conclusion and future directions

This peripheral phenotyping study, integrating protein and cell analysis with dimensional and feature selection approaches, identified immune-inflammatory alterations in MDE, irrespective of recent SA. Specifically, we observed reduced immune modulation proteins and growth factors, a shift toward innate immune dominance over adaptive immunity, and subtle neuroinflammatory changes, consistent with prior evidence linking immune dysregulation to depression. However, no robust immune-inflammatory differences were found between MDE patients with and without recent SA, possibly due to post-crisis immune normalization, sample size limitations, or the heterogeneity of SA itself. While higher NLR and lower serotonin levels differentiated SA patients from healthy controls, no single marker consistently distinguished MDE patients with SA from those without SA history. These findings underscore the need for larger, longitudinal studies to track immune changes before and after SA and to disentangle state- and trait-related signatures. Given the emerging role of monocyte subsets, T-cell exhaustion markers, and neuroimmune interactions in MDE and SA, future research should integrate deeper immune phenotyping. Additionally, further exploration of symptom-specific immune correlates is warranted, considering the heterogeneity of MDE and the complex interplay between inflammation, depression, and suicidal behaviors.

## Supplementary information

Supplementary figure and table legends

Supplemental Table S1

Supplemental Table S2

Supplemental Table S3

Supplemental Table S4

Supplemental Table S5

Supplemental Table S6

Supplemental Figure S1

Supplemental Figure S2

Supplemental Figure S3

Supplemental Figure S4

Supplemental Figure S5

Supplemental Figure S6

Supplemental table S7

## Data Availability

The data are subject to national data protection laws. Therefore, data cannot be made freely available in a public repository. However, data can be requested through a reasonable requesto the the corresponding author.
