# Supplementary material for: Integrating cellular and soluble immune signatures of major depression with and without recent suicide attempts
Source: Transl Psychiatry. 2025 Oct 6;15:377. doi: 10.1038/s41398-025-03601-2 (PMC12501231; doi:10.1038/s41398-025-03601-2)
Supplement: Supplementary file 1 — Supplementary figure and table legends [file 41398_2025_3601_MOESM1_ESM.docx]

Table S1: Univariate differences between HC and AC. Mean (± sd) and N (%) are presented for quantitative and qualitative variables. F-test or Pearson’s chi-squared test are used according to the variable type. P-values are not corrected for multiple testing.

Table S2: Univariate differences between HC and SA. Mean (± sd) and N (%) are presented for quantitative and qualitative variables. F-test or Pearson’s chi-squared test are used according to the variable type. P-values are not corrected for multiple testing.

Table S3: Univariate differences between AC and SA. Mean (± sd) and N (%) are presented for quantitative and qualitative variables. F-test or Pearson’s chi-squared test are used according to the variable type. P-values are not corrected for multiple testing.

Table S4. Univariate pairwise comparisons between people with depression with a recent suicide attempt and healthy controls.

Table S5. Supplemental Table S5. Univariate pairwise comparisons between people with major depressive episode with and without a recent suicide attempt. Non-adjusted pairwise comparisons. P-values are two-sided. MDE, major depressive episode; SA, suicide attempters.

Table S6. Multivariate associations between study groups and MFA dimensions. Abbreviations: LRT, likelihood ratio test; MDE, major depressive disorder, without suicide attempt history; HC, healthy controls; OR, Odds Ratio; SA, suicide attempters. Multivariate associations are estimated from logistic regression, adjusted for sex and age.

Table S7. Multivariate associations between study groups and MFA dimensions in a subgroup of patients with major depressive disorder. Abbreviations: LRT, likelihood ratio test; MDE, major depressive disorder, without suicide attempt history; HC, healthy controls; OR, Odds Ratio; SA, suicide attempters. Multivariate associations are estimated from logistic regression, adjusted for sex and age.

Figure S1: Odds Ratio and 95% confidence intervals estimated from multivariate logistic regression contrasting HC and AC groups of subjects, according to the three methods of variable selection used (sequential Step AIC, LASSO and SSVS). Potential confounding factors, sex and age are included in models.

Figure S2: Odds Ratio and 95% confidence intervals estimated from multivariate logistic regression contrasting HC and SA groups of subjects, according to the three methods of variable selection used (sequential Step AIC, LASSO and SSVS). Potential confounding factors, sex and age are included in models.

Figure S3: Odds Ratio and 95% confidence intervals estimated from multivariate logistic regression contrasting AC and SA groups of subjects, according to the three methods of variable selection used (sequential Step AIC, LASSO and SSVS). Potential confounding factors, sex and age are included in models.

Figure S4. Representation of individuals across the first three Multiple Factor Analysis dimensions by study group in a subsample of patients with major depressive disorder.

Figure S5. Characterization of the three meaningful Multiple Factor analysis dimensions in a subsample of patients with major depressive disorder.
