# Supplementary material for: Integrating cellular and soluble immune signatures of major depression with and without recent suicide attempts
Source: Transl Psychiatry. 2025 Oct 6;15:377. doi: 10.1038/s41398-025-03601-2 (PMC12501231; doi:10.1038/s41398-025-03601-2)
Supplement: Supplementary file 2 — Supplemental Table S1 [file 41398_2025_3601_MOESM2_ESM.docx]

Supplemental Table S1. Platforms and kits used for protein analysis

| Variable | Abbreviation | Platform | Kit |
| --- | --- | --- | --- |
| Interferon gamma, pg/ml | IFN-$\gamma$ | Merck Millipore | Plex HCYTOMAG-60K |
| Interleukin-6, pg/ml | IL-6 | Merck Millipore | Plex HCYTOMAG-60K |
| Interleukin-1 beta, pg/ml | IL-1$\beta$ | Merck Millipore | Plex HCYTOMAG-60K |
| Tumor necrosis factor alpha, pg/ml | TNF-$\alpha$ | Merck Millipore | Plex HCYTOMAG-60K |
| Interleukin 4, pg/ml | IL-4 | Merck Millipore | Plex HCYTOMAG-60K |
| Regulated on activation, normal T cell expressed and secreted, pg/ml | RANTES | Bio-Techne SA | ref DY278 |
| Thrombospondin-1, ng/ml | TSP-1 | Bio-Techne SA | ref DY1635 |
| Thrombospondin-2, ng/ml | TSP-2 | Bio-Techne SA | ref DY3074 |
| Platelet-derived growth factor-AB, pg/ml | PDGF-AB | Bio-Techne SA | ref DY222 |
| Platelet-derived growth factor-BB, pg/ml | PDGF-BB | Bio-Techne SA | ref DY220 |
| Transforming growth factor beta 1, pg/ml | TGF1- $\beta$1 | Bio-Techne SA | ref DY240 |
| Monocyte chemoattractant protein-1 |  | Bio-Techne SA | ref DCP00 |
| Serotonin ng/ml | 5-HT | Enzo Life Sciences | ADI-900-175 |
| Annexin ng/ml | ANXA | Bio-Techne SA | DY2900-05 |
| Uteroglobin ng/ml | CC16 | BioVendor | RD191022200 |
| Centrin ng/ml | CETN1 | Biomatik | ABIN420901 |
| Glial fibrillary acidic protein pg/ml | GFAP | Quanterix (Simoa platform) | 103520 |
| Neurofilament light chain pg/ml | NFL | Quanterix (Simoa platform) | 103186 |
