# Supplementary material for: Integrating cellular and soluble immune signatures of major depression with and without recent suicide attempts
Source: Transl Psychiatry. 2025 Oct 6;15:377. doi: 10.1038/s41398-025-03601-2 (PMC12501231; doi:10.1038/s41398-025-03601-2)
Supplement: Supplementary file 3 — Supplemental Table S2 [file 41398_2025_3601_MOESM3_ESM.docx]

Supplemental Table S2. Numbers of undetectable and missing values

| Variable | Undetectable values N (%) | Imputation method | Missing values N (%) | Imputation method |
| --- | --- | --- | --- | --- |
| Interferon gamma | 53 (50%) | Categorical (2 groups) | 0 (0%) | .. |
| Interleukin-6 | 26 (24.5%) | Categorical (3 groups) | 0 (0%) | .. |
| Interleukin-1 beta | 33 (31.1%) | Categorical (2 groups) | 0 (0%) | .. |
| Tumor necrosis factor alpha | 28 (26.4%) | Categorical (3 groups) | 0 (0%) | .. |
| Interleukin 4 | 12 (11.3%) | Log-normal | 0 (0%) | .. |
| Regulated on activation, normal T cell expressed and secreted | 11 (10.4%) | Categorical (3 groups) | 0 (0%) | .. |
| Thrombospondin-1 | 0 (0%) | .. | 0 (0%) | .. |
| Thrombospondin-2 | 0 0%) | .. | 0 (0%) | .. |
| Platelet-derived growth factor-AB | 34 (32.1%) | Categorical (3 groups) | 0 (0%) | .. |
| Platelet-derived growth factor-BB | 9 (8.5%) | Log-normal | 0 (0%) | .. |
| Transforming growth factor beta 1 | 0 (0%) | .. | 0 (0%) | .. |
| Serotonin | 0 (0%) | .. | 0 (0%) | .. |
| Annexin | 0 (0%) | .. | 0 (0%) | .. |
| Uteroglobin | 19 (17.9%) | Categorical (3 groups) | 0 (0%) | .. |
| Centrin | 0 (0%) | .. | 0 (0%) | .. |
| Monocyte chemoattractant protein-1 | 0 (0%) | .. | 0 (0%) | .. |
| Glial fibrillary acidic protein | 0 (0%) | .. | 0 (0%) | .. |
| Neurofilament light chain | 0 (0%) | .. | 0 (0%) | .. |
| CD3% in single cells | 0 (0%) | .. | 6 (5.7%) | MAF |
| CD3% in CD45 | 0 (0%) | .. | 5 (4.7%) | MAF |
| CD14% in CD45 | 0 (0%) | .. | 6 (5.7%) | MAF |
| CD4% in CD3 | 0 (0%) | .. | 6 (5.7%) | MAF |
| CD8% in CD3 | 0 (0%) | .. | 7 (6.6%) | MAF |
| CD4/CD8 ratio | 0 (0%) | .. | 7 (6.6%) | MAF |
| Eosinophil count | 0 (0%) | .. | 3 (2.8%) | MAF |
| Basophil count | 0 (0%) |  | 3 (2.8%) | MAF |
| Neutrophil-to-lyphocyte ratio | 0 (0%) | .. | 3 (2.8%) | MAF |
| Monocyte-to-lymphocyte ratio | 0 (0%) | .. | 3 (2.8%) | MAF |
| Platelet-to-lymphocyte ratio | 0 (0%) | .. | 3 (2.8%) | MAF |
| C-reactive protein | 0 (0%) | .. | 0 (0%) | .. |
