# Supplementary material for: Integrating cellular and soluble immune signatures of major depression with and without recent suicide attempts
Source: Transl Psychiatry. 2025 Oct 6;15:377. doi: 10.1038/s41398-025-03601-2 (PMC12501231; doi:10.1038/s41398-025-03601-2)
Supplement: Supplementary file 4 — Supplemental Table S3 [file 41398_2025_3601_MOESM4_ESM.docx]

Supplemental Table S3. Univariate pairwise comparisons between major depressive episode without suicide attempt history and healthy controls

| **Variable** | **Level** | **HC**  **Mean (s.d.) or N (%)** | **MDE without SA**  **Mean (s.d.) or N (%)** | **P-value** | **Statistics** | **Missing** |
| --- | --- | --- | --- | --- | --- | --- |
| Sex | Men | 9( 25.7) | 9 (25.7) | 1 | Chi^2^ = 0 ; df = 1 | 0 |
|  | Women | 26 (74.3) | 26 (74.3) | NA | NA |  |
| Age |  | 34.114 (10.701) | 33.706 (10.842) | 0.88 | F = 0.025 ; df = 1 ; dfres = 67 | 1 |
| Metabolic syndrome | No | 34 (97.1) | 32 (91.4) | 0.61 | Chi^2^ = NA ; df = NA | 0 |
|  | Yes | 1 (2.9) | 3 (8.6) | NA | NA |  |
| Tobacco user | Non-user | 27 (77.1) | 20 (57.1) | 0.13 | Chi^2^ = 2.331 ; df = 1 | 0 |
|  | User | 8 (22.9) | 15 (42.9) | NA | NA |  |
| Nicotine intake | No | 27 (77.1) | 19 (54.3) | 0.08 | Chi^2^ = 3.107 ; df = 1 | 0 |
|  | Yes | 8 (22.9) | 16 (45.7) | NA | NA |  |
| BDI total score |  | 0.829 (1.317) | 17.429 (7.942) | <0.0001 | F = 148.813 ; df = 1 ; dfres = 68 | 0 |
| Current suicidal ideation (BDI) | No | 35 (100) | 30 (85.7) | 0.05 | Chi^2^ = NA ; df = NA | 0 |
|  | Yes | 0 (0) | 5 (14.3) | NA | NA |  |
| IFN-$\gamma$ | Undetected | 12 (34.3) | 24 (68.6) | 0.009 | Chi^2^ = 6.92 ; df = 1 | 0 |
|  | Detected | 23 (65.7) | 11 (31.4) | NA | NA |  |
| IL-1$\beta$ | Undetected | 7 (20) | 17 (48.6) | 0.02 | Chi^2^ = 5.136 ; df = 1 | 0 |
|  | Detected | 28 (80) | 18 (51.4) | NA | NA |  |
| IL-2 | Undetected | 14 (40) | 25 (71.4) | 0.02 | Chi^2^ = 5.79 ; df = 1 | 0 |
|  | Detected | 21 (60) | 10 (28.6) | NA | NA |  |
| IL-6 tercile | Ter 1 | 6 (17.1) | 11 (31.4) | 0.18 | Chi^2^ = 3.439 ; df = 2 | 0 |
|  | Ter 2 | 12 (34.3) | 14 (40) | NA | NA |  |
|  | Ter 3 | 17 (48.6) | 10 (28.6) | NA | NA |  |
| TNF-$\alpha$ tercile | Ter 1 | 7 (20) | 14 (40) | 0.13 | Chi^2^ = 4.007 ; df = 2 | 0 |
|  | Ter 2 | 14 (40) | 13 (37.1) | NA | NA |  |
|  | Ter 3 | 14 (40) | 8 (22.9) | NA | NA |  |
| PDGF-AB tercile | Ter 1 | 7 (20) | 20 (57.1) | 0.003 | Chi^2^ = 11.321 ; df = 2 | 0 |
|  | Ter 2 | 10 (28.6) | 8 (22.9) | NA | NA |  |
|  | Ter 3 | 18 (51.4) | 7 (20) | NA | NA |  |
| RANTES tertile | Ter 1 | 6 (17.1) | 16 (45.7) | 0.0002 | Chi^2^ = 17.582 ; df = 2 | 0 |
|  | Ter 2 | 7 (20) | 14 (40) | NA | NA |  |
|  | Ter 3 | 22 (62.9) | 5 (14.3) | NA | NA |  |
| Uteroglobin 3c tercile | Ter 1 | 16 (45.7) | 13 (37.1) | 0.34 | Chi^2^ = 2.131 ; df = 2 | 0 |
|  | Ter 2 | 14 (40) | 12 (34.3) | NA | NA |  |
|  | Ter 3 | 5 (14.3) | 10 (28.6) | NA | NA |  |
| IL-4 log (imp) |  | 2.136 (1.41) | 1.843 (1.416) | 0.39 | F = 0.755 ; df = 1 ; dfres = 68 | 0 |
| Annexin log (imp) |  | -0.693 (1.528) | -0.274 (1.701) | 0.28 | F = 1.178 ; df = 1 ; dfres = 68 | 0 |
| PDGF-BB log (imp) |  | 5.804 (1.037) | 4.675 (1.473) | 0.0004 | F = 13.744 ; df = 1 ; dfres = 68 | 0 |
| CRP log (imp) |  | 0.088 (1.021) | 0.037 (0.997) | 0.83 | F = 0.046 ; df = 1 ; dfres = 68 | 0 |
| TSP- 2 log (imp) |  | 10.152 (0.294) | 10.095 (0.3) | 0.42 | F = 0.654 ; df = 1 ; dfres = 68 | 0 |
| MCP-1 log (imp) |  | 5.41 (0.464) | 5.421 (0.484) | 0.92 | F = 0.01 ; df = 1 ; dfres = 68 | 0 |
| Serotonine log (imp) |  | 3.057 (0.612) | 2.369 (0.345) | <0.0001 | F = 33.534 ; df = 1 ; dfres = 68 | 0 |
| TGF-1$\beta$ log (imp) |  | 5.155 (0.536) | 4.677 (0.476) | 0.0002 | F = 15.518 ; df = 1 ; dfres = 68 | 0 |
| TSP-1 log (imp) |  | 12.48 (0.522) | 12.085 (0.718) | 0.01 | F = 6.923 ; df = 1 ; dfres = 68 | 0 |
| Centrin 2 log (imp) |  | -0.452 (0.939) | -0.609 (0.687) | 0.43 | F = 0.632 ; df = 1 ; dfres = 68 | 0 |
| GFAP (log) |  | 4.184 (0.459) | 4.35 (0.34) | 0.09 | F = 2.928 ; df = 1 ; dfres = 68 | 0 |
| NFL (log) |  | 1.99 (0.408) | 2.155 (0.504) | 0.14 | F = 2.279 ; df = 1 ; dfres = 68 | 0 |
| CD3% in single cells |  | 23.444 (5.717) | 20.002 (5.001) | 0.01 | F = 6.801 ; df = 1 ; dfres = 64 | 4 |
| CD3% in single cells (imp) |  | 23.571 (5.482) | 19.89 (4.971) | 0.004 | F = 8.658 ; df = 1 ; dfres = 68 | .. |
| CD3% in CD45 |  | 68.309(6.752) | 69.913 (7.342) | 0.36 | F = 0.851 ; df = 1 ; dfres = 64 | 4 |
| CD3% in CD45 (imp) |  | 68.332 (6.618) | 69.399 (7.847) | 0.54 | F = 0.378 ; df = 1 ; dfres = 68 | .. |
| CD14% in CD45 |  | 7.57 (2.964) | 7.988 (4.833) | 0.68 | F = 0.173 ; df = 1 ; dfres = 63 | 5 |
| CD14% in CD45 (imp) |  | 7.692 (2.905) | 8.104 (4.81) | 0.67 | F = 0.188 ; df = 1 ; dfres = 68 | .. |
| CD4% in CD3 |  | 55.101 (9.775) | 61.543 (9.125) | 0.007 | F = 7.668 ; df = 1 ; dfres = 64 | 4 |
| CD4% in CD3 (imp) |  | 55.57 (9.679) | 61.425 (9.016) | 0.01 | F = 6.858 ; df = 1 ; dfres = 68 | .. |
| CD8% in CD3 |  | 36.297 (6.887) | 29.516 (7.991) | 0.0005 | F = 13.307 ; df = 1 ; dfres = 63 | 5 |
| CD8% in CD3 (imp) |  | 35.623 (7.026) | 29.693 (7.941) | 0.002 | F = 10.946 ; df = 1 ; dfres = 68 | .. |
| CD4/CD8 ratio |  | 1.603 (0.529) | 2.35 (1.225) | 0.003 | F = 9.848 ; df = 1 ; dfres = 63 | 5 |
| Nucleated cell count |  | 6.308 (1.758) | 6.862 (1.56) | 0.18 | F = 1.86 ; df = 1 ; dfres = 65 | 3 |
| Platelet count |  | 249.629 (64.897) | 248.118 (55.868) | 0.92 | F = 0.011 ; df = 1 ; dfres = 67 | 1 |
| Neutrophil count |  | 3.665 (1.253) | 4.163 (1.242) | 0.11 | F = 2.669 ; df = 1 ; dfres = 65 | 3 |
| Eosinophil count |  | 0.186 (0.127) | 0.181 (0.115) | 0.84 | F = 0.04 ; df = 1 ; dfres = 65 | 3 |
| Eosinophil count (imp) |  | 0.185 (0.125) | 0.186 (0.114) | 0.96 | F = 0.002 ; df = 1 ; dfres = 68 | .. |
| Basophil count |  | 0.042 (0.015) | 0.052 (0.024) | 0.06 | F = 3.537 ; df = 1 ; dfres = 65 | 3 |
| Basophil count (imp) |  | 0.043 (0.015) | 0.052 (0.023) | 0.048 | F = 4.045 ; df = 1 ; dfres = 68 | .. |
| Lymphocyte cunt |  | 1.965 (0.623) | 1.987 (0.546) | 0.88 | F = 0.022 ; df = 1 ; dfres = 65 | 3 |
| Monocyte count |  | 0.449 (0.146) | 0.481 (0.132) | 0.35 | F = 0.873 ; df = 1 ; dfres = 65 | 3 |
| Blood NLR |  | 1.96 (0.748) | 2.22 (0.813) | 0.18 | F = 1.853 ; df = 1 ; dfres = 65 | 3 |
| Blood NLR (imp) |  | 1.985 (0.751) | 2.195 (0.811) | 0.26 | F = 1.265 ; df = 1 ; dfres = 68 | .. |
| Blood PLR |  | 140.079 (53.653) | 131.293 (40.625) | 0.45 | F = 0.569 ; df = 1 ; dfres = 65 | 3 |
| Blood PLR (imp) |  | 140.818 (53.038) | 131.337 (39.558) | 0.4 | F = 0.719 ; df = 1 ; dfres = 68 | .. |
| Blood MLR |  | 0.243 (0.09) | 0.25 (0.068) | 0.7 | F = 0.151 ; df = 1 ; dfres = 65 | 3 |
| Blood MLR (imp) |  | 0.244 (0.089) | 0.248 (0.07) | 0.87 | F = 0.027 ; df = 1 ; dfres = 68 | .. |
| MFA Dim 1 |  | 0.775 (1.357) | -0.667 (1.062) | <0.0001 | F = 24.536 ; df = 1 ; dfres = 68 | 0 |
| MFA Dim 2 |  | -0.558 (0.963) | 0.204 (1.14) | 0.004 | F = 9.138 ; df = 1 ; dfres = 68 | 0 |
| MFA Dim 3 |  | -0.177 (1.036) | 0.125 (0.941) | 0.21 | F = 1.635 ; df = 1 ; dfres = 68 | 0 |
| Treatment |  |  |  |  |  |  |
| Anxiolytic or hypnotic | No | 35 (100) | 13 (37.1) | <0.0001 | Chi^2^ = 29.233; df = 1 | 0 |
|  | Yes | 0 (0) | 22 (62.9) | NA | NA |  |
| Antidepressant | No | 35 (100) | 6 (17.1) | <0.0001 | Chi^2^ = 46.156; df = 1 | 0 |
|  | Yes | 0 (0) | 29 (82.9) | NA | NA |  |
| Antipsychotic | No | 35 (100) | 21 (60) | 0.0001 | Chi^2^ = 15.089; df = 1 | 0 |
|  | Yes | 0 (0) | 14 (40) | NA | NA |  |
| Mood stabilizer | No | 35 (100) | 21 (60) | 0.0001 | Chi^2^ = 15.089 ; df = 1 | 0 |
|  | Yes | 0 (0) | 14 (40) | NA | NA |  |

Non-adjusted pairwise comparisons. P-values are two-sided.

MDE without SA, major depressive episode without suicide attempt history; HC, healthy controls.
