# Supplementary material for: Integrating cellular and soluble immune signatures of major depression with and without recent suicide attempts
Source: Transl Psychiatry. 2025 Oct 6;15:377. doi: 10.1038/s41398-025-03601-2 (PMC12501231; doi:10.1038/s41398-025-03601-2)
Supplement: Supplementary file 5 — Supplemental Table S4 [file 41398_2025_3601_MOESM5_ESM.docx]

Supplemental Table S4. Univariate pairwise comparisons between people with depression with a recent suicide attempt and healthy controls

| variable | Level | HC  Mean (s.d.) or N (%) | SA  Mean (s.d.) or N (%) | P-value | Statistics | Missing |
| --- | --- | --- | --- | --- | --- | --- |
| Sex | Men | 9 (25.7) | 9 (25) | 1 | Chi^2^ = 0 ; df = 1 | 0 |
|  | Women | 26 (74.3) | 27 (75) | NA | NA |  |
| Age |  | 34.114 (10.701) | 32.167 (12.852) | 0.49 | F = 0.48 ; df = 1 ; dfres = 69 | 0 |
| Metabolic syndrome | No | 34 (97.1) | 33 (91.7) | 0.61 | Chi^2^ = NA ; df = NA | 0 |
|  | Yes | 1 (2.9) | 3 (8.3) | NA | NA |  |
| Tobacco user | Non-user | 27 (77.1) | 22 (61.1) | 0.23 | Chi^2^ = 1.449 ; df = 1 | 0 |
|  | User | 8 (22.9) | 14 (38.9) | NA | NA |  |
| Nicotine intake | No | 27 (77.1) | 20 (55.6) | 0.09 | Chi^2^ = 2.794 ; df = 1 | 0 |
|  | Yes | 8 (22.9) | 16 (44.4) | NA | NA |  |
| BDI total score |  | 0.829 (1.317) | 20.556 (6.097) | <0.0001 | F = 350.42 ; df = 1 ; dfres = 69 | 0 |
| Current suicidal ideation (BDI) | No | 35 (100) | 28 (77.8) | 0.005 | Chi^2^ = NA ; df = NA | 0 |
|  | Yes | 0 (0) | 8 (22.2) | NA | NA |  |
| IFN-$\boldsymbol{\gamma}$ | Undetected | 12 (34.3) | 21 (58.3) | 0.07 | Chi^2^ = 3.215 ; df = 1 | 0 |
|  | Detected | 23 (65.7) | 15 (41.7) | NA | NA |  |
| IL-1$\boldsymbol{\beta}$ | Undetected | 7 (20) | 9 (25) | 0.83 | Chi^2^ = 0.048 ; df = 1 | 0 |
|  | Detected | 28 (80) | 27 (75) | NA | NA |  |
| IL-2 | Undetected | 14 (40) | 22 (61.1) | 0.12 | Chi^2^ = 2.376 ; df = 1 | 0 |
|  | Detected | 21 (60) | 14 (38.9) | NA | NA |  |
| IL-6 tertile | Ter 1 | 6 (17.1) | 9 (25) | 0.41 | Chi^2^ = 1.782 ; df = 2 | 0 |
|  | Ter 2 | 12 (34.3) | 15 (41.7) | NA | NA |  |
|  | Ter 3 | 17 (48.6) | 12 (33.3) | NA | NA |  |
| TNF-$\boldsymbol{\alpha}$ tertile | Ter 1 | 7 (20) | 7 (19.4) | 0.66 | Chi^2^ = 0.846 ; df = 2 | 0 |
|  | Ter 2 | 14 (40) | 11 (30.6) | NA | NA |  |
|  | Ter 3 | 14 (40) | 18 (50) | NA | NA |  |
| PDGF-AB tertile | Ter 1 | 7 (20) | 11 (30.6) | 0.04 | Chi^2^ = 6.537 ; df = 2 | 0 |
|  | Ter 2 | 10 (28.6) | 17 (47.2) | NA | NA |  |
|  | Ter 3 | 18 (51.4) | 8 (22.2) | NA | NA |  |
| RANTES tertile | Ter 1 | 6 (17.1) | 14 (38.9) | 0.002 | Chi^2^ = 12.055 ; df = 2 | 0 |
|  | Ter 2 | 7 (20) | 14 (38.9) | NA | NA |  |
|  | Ter 3 | 22 (62.9) | 8 (22.2) | NA | NA |  |
| Uteroglobin 3c tertile | Ter 1 | 16 (45.7) | 15 (41.7) | 0.86 | Chi^2^ = NA ; df = NA | 0 |
|  | Ter 2 | 14 (40) | 17 (47.2) | NA | NA |  |
|  | Ter 3 | 5 (14.3) | 4 (11.1) | NA | NA |  |
| IL-4 log (imp) |  | 2.136 (1.41) | 2.062 (1.552) | 0.83 | F = 0.044 ; df = 1 ; dfres = 69 | 0 |
| Annexin log |  | -0.693 (1.528) | -0.354 (1.746) | 0.39 | F = 0.758 ; df = 1 ; dfres = 69 |  |
| PDGF-BB log |  | 5.804 (1.037) | 4.922 (1.051) | 0.0007 | F = 12.656 ; df = 1 ; dfres = 69 | 0 |
| CRP log |  | 0.088 (1.021) | 0.07 (0.906) | 0.93 | F = 0.007 ; df = 1 ; dfres = 69 | 0 |
| TSP- 2 log |  | 10.152 (0.294) | 10.1 (0.235) | 0.41 | F = 0.688 ; df = 1 ; dfres = 69 | 0 |
| MCP-1 log |  | 5.41 (0.464) | 5.501 (0.421) | 0.39 | F = 0.754 ; df = 1 ; dfres = 69 | 0 |
| Serotonin log |  | 3.057 (0.612) | 2.484 (0.519) | <0.0001 | F = 18.145 ; df = 1 ; dfres = 69 | 0 |
| TGF-1$\boldsymbol{\beta}$ log |  | 5.155 (0.536) | 4.723 (0.402) | 0.0003 | F = 14.843 ; df = 1 ; dfres = 69 | 0 |
| TSP-1 log |  | 12.48 (0.522) | 12.052 (0.655) | 0.003 | F = 9.216 ; df = 1 ; dfres = 69 | 0 |
| Centrin 2 log |  | -0.452 (0.939) | -0.795 (0.664) | 0.08 | F = 3.17 ; df = 1 ; dfres = 69 | 0 |
| GFAP tertile | Ter 1 | 15 (42.9) | 12 (34.3) | 0.53 | Chi^2^ = 1.262 ; df = 2 | 0 |
|  | Ter 2 | 7 (20) | 11 (31.4) | NA | NA |  |
|  | Ter 3 | 13 (37.1) | 12 (34.3) | NA | NA |  |
| GFAP log |  | 4.184 (0.459) | 4.263 (0.488) | 0.49 | F = 0.488 ; df = 1 ; dfres = 69 | 0 |
| NFL tercile | Ter 1 | 16 (45.7) | 9 (25.7) | 0.21 | Chi^2^ = 3.12 ; df = 2 | 0 |
|  | Ter 2 | 11 (31.4) | 14 (40) | NA | NA |  |
|  | Ter 3 | 8 (22.9) | 12 (34.3) | NA | NA |  |
| NFL log |  | 1.99 (0.408) | 2.155 (0.57) | 0.17 | F = 1.938 ; df = 1 ; dfres = 68 | 0 |
| CD3% in single cells |  | 23.444 (5.717) | 20.46 (6.629) | 0.06 | F = 3.768 ; df = 1 ; dfres = 63 | 6 |
| CD3% in single cells (imp) |  | 23.571 (5.482) | 20.411 (6.387) | 0.03 | F = 4.989 ; df = 1 ; dfres = 69 |  |
| CD3% in CD45 |  | 68.309 (6.752) | 68.893 (9.943) | 0.78 | F = 0.077 ; df = 1 ; dfres = 64 | 5 |
| CD3% in CD45 (imp) |  | 68.332 (6.618) | 69.012 (9.705) | 0.73 | F = 0.118 ; df = 1 ; dfres = 69 |  |
| CD14% in CD45 |  | 7.57 (2.964) | 7.785 (4.748) | 0.83 | F = 0.047 ; df = 1 ; dfres = 63 | 6 |
| CD14% in CD45 (imp) |  | 7.692 (2.905) | 7.737 (4.63) | 0.96 | F = 0.002 ; df = 1 ; dfres = 69 |  |
| CD4% in CD3 |  | 55.101 (9.775) | 57.077 (10.516) | 0.44 | F = 0.614 ; df = 1 ; dfres = 63 | 5 |
| CD4% in CD3 (imp) |  | 55.57 (9.679) | 57.186 (10.139) | 0.49 | F = 0.472 ; df = 1 ; dfres = 69 | .. |
| CD8% in CD3 |  | 36.297 (6.887) | 32.614 (7.897) | 0.05 | F = 3.934 ; df = 1 ; dfres = 62 | 6 |
| CD8% in CD3 (imp) |  | 35.623 (7.026) | 32.707 (7.614) | 0.1 | F = 2.809 ; df = 1 ; dfres = 69 | .. |
| CD4/CD8 ratio |  | 1.603 (0.529) | 1.895 (0.679) | 0.06 | F = 3.674 ; df = 1 ; dfres = 62 | 6 |
| Nucleated cell count |  | 6.308 (1.758) | 7.292 (2.119) | 0.041 | F = 4.346 ; df = 1 ; dfres = 66 | 3 |
| Platelet count |  | 249.629 (64.897) | 253.657 (71.408) | 0.81 | F = 0.061 ; df = 1 ; dfres = 68 | 1 |
| Neutrophil count |  | 3.665 (1.253) | 4.689 (1.974) | 0.01 | F = 6.532 ; df = 1 ; dfres = 66 | 3 |
| Eosinophil count |  | 0.186 (0.127) | 0.187 (0.136) | 0.99 | F = 0 ; df = 1 ; dfres = 66 | 3 |
| Eosinophil count (imp) |  | 0.185 (0.125) | 0.181 (0.137) | 0.89 | F = 0.02 ; df = 1 ; dfres = 69 | .. |
| Basophil count |  | 0.042 (0.015) | 0.053 (0.019) | 0.01 | F = 6.315 ; df = 1 ; dfres = 66 | 3 |
| Basophil count (imp) |  | 0.043 (0.015) | 0.052 (0.019) | 0.02 | F = 5.458 ; df = 1 ; dfres = 69 | .. |
| Lymphocyte cunt |  | 1.965 (0.623) | 1.861 (0.498) | 0.45 | F = 0.58 ; df = 1 ; dfres = 66 | 3 |
| Monocyte count |  | 0.449 (0.146) | 0.502 (0.171) | 0.17 | F = 1.888 ; df = 1 ; dfres = 66 | 3 |
| Blood NLR |  | 1.96 (0.748) | 2.714 (1.353) | 0.006 | F = 8.071 ; df = 1 ; dfres = 66 | 3 |
| Blood NLR (imp) |  | 1.985 (0.751) | 2.707 (1.314) | 0.006 | F = 8.02 ; df = 1 ; dfres = 69 | .. |
| Blood PLR |  | 140.079 (53.653) | 143.38 (39.58) | 0.77 | F = 0.083 ; df = 1 ; dfres = 66 | 3 |
| Blood PLR (imp) |  | 140.818 (53.038) | 141.371 (39.358) | 0.96 | F = 0.002 ; df = 1 ; dfres = 69 | .. |
| Blood MLR |  | 0.243 (0.09) | 0.293 (0.154) | 0.1 | F = 2.728 ; df = 1 ; dfres = 66 | 3 |
| Blood MLR (imp) |  | 0.244 (0.089) | 0.293 (0.15) | 0.1 | F = 2.747 ; df = 1 ; dfres = 69 | .. |
| MFA Dim 1 |  | 0.775 (1.357) | -0.105 (1.335) | 0.008 | F = 7.584 ; df = 1 ; dfres = 69 | .. |
| MFA Dim 2 |  | -0.558 (0.963) | 0.344 (1.407) | 0.002 | F = 9.898 ; df = 1 ; dfres = 69 | .. |
| MFA Dim 3 |  | -0.177 (1.036) | 0.051 (1.307) | 0.42 | F = 0.662 ; df = 1 ; dfres = 69 | .. |
| Treatment |  |  |  |  |  |  |
| Anxiolytic or hypnotic | No | 35 (100) | 4 (11.1) | <0.0001 | Chi^2^ = 53.105 ; df = 1 | 0 |
|  | Yes | 0 (0) | 32 (88.9) | NA | NA |  |
| Antidepressant | No | 35 (100) | 10 (27.8) | <0.0001 | Chi^2^ = 36.832 ; df = 1 | 0 |
|  | Yes | 0 (0) | 26 (72.2) | NA | NA |  |
| Antipsychotic | No | 35 (100) | 14 (38.9) | <0.0001 | Chi^2^ = 28.2 ; df = 1 | 0 |
|  | Yes | 0 (0) | 22 (61.1) | NA | NA |  |
| Mood stabilizer | No | 35 (100) | 14 (38.9) | <0.0001 | Chi^2^ = 28.2 ; df = 1 | 0 |
|  | Yes | 0 (0) | 22 (61.1) | NA | NA |  |

Non-adjusted pairwise comparisons. P-values are two-sided.
