# Supplementary material for: Integrating cellular and soluble immune signatures of major depression with and without recent suicide attempts
Source: Transl Psychiatry. 2025 Oct 6;15:377. doi: 10.1038/s41398-025-03601-2 (PMC12501231; doi:10.1038/s41398-025-03601-2)
Supplement: Supplementary file 6 — Supplemental Table S5 [file 41398_2025_3601_MOESM6_ESM.docx]

Supplemental Table S5. Univariate pairwise comparisons between people with major depressive episode with and without a recent suicide attempt

| **Variable** | **Level** | **MDE without SA**  **Mean (s.d.) or N (%)** | **SA**  **Mean (s.d.) or N (%)** | **P-value** | **Statistics** | **Missing** |
| --- | --- | --- | --- | --- | --- | --- |
| Sex | Men | 9 (25.7) | 9 (25) | 1 | Chi^2^ = 0 ; df = 1 | 0 |
|  | Women | 26 (74.3) | 27 (75) | NA | NA |  |
| Age |  | 33.706 (10.842) | 32.167 (12.852) | 0.59 | F = 0.292 ; df = 1 ; dfres = 68 | 0 |
| Metabolic syndrome | No | 32 (91.4) | 33 (91.7) | 1 | Chi^2^= NA ; df = NA | 0 |
|  | Yes | 3 (8.6) | 3 (8.3) | NA | NA |  |
| Tobacco user | Non user | 20 (57.1) | 22 (61.1) | 0.92 | Chi^2^ = 0.01 ; df = 1 | 0 |
|  | User | 15 (42.9) | 14 (38.9) | NA | NA |  |
| Nicotine intake | No | 19 (54.3) | 20 (55.6) | 1 | Chi^2^ = 0 ; df = 1 | 0 |
|  | Yes | 16 (45.7) | 16 (44.4) | NA | NA |  |
| Recurrent major depressive disorder | No | 14 (40) | 15 (41.7) | 1 | Chi^2^ = 0 ; df = 1 | 0 |
|  | Yes | 21 (60) | 21 (58.3) | NA | NA |  |
| Lifetime depressive disorder | No | 7 (20) | 5 (13.9) | 0.71 | Chi^2^ = 0.137 ; df = 1 | 0 |
|  | Yes | 28 (80) | 31 (86.1) | NA | NA |  |
| Bipolar disorder | No | 29 (82.9) | 31 (86.1) | 0.96 | Chi^2^ = 0.003 ; df = 1 | 0 |
|  | Yes | 6 (17.1) | 5 (13.9) | NA | NA |  |
| Type 1 bipolar disorder | No | 33 (94.3) | 35 (97.2) | 0.61 | Chi^2^ = NA ; df = NA | 0 |
|  | Yes | 2 (5.7) | 1 (2.8) | NA | NA |  |
| Type 2 bipolar disorder | No | 31 (88.6) | 32 (88.9) | 1 | Chi^2^ = NA ; df = NA | 0 |
|  | Yes | 4 (11.4) | 4 (11.1) | NA | NA |  |
| Anxiety disorder, lifetime | No | 11 (31.4) | 16 (44.4) | 0.38 | Chi^2^ = 0.783 ; df = 1 | 0 |
|  | Yes | 24 (68.6) | 20 (55.6) | NA | NA |  |
| Alcohol or substance use disorder, lifetime | No | 29 (82.9) | 27 (75) | 0.6 | Chi^2^ = 0.27 ; df = 1 | 0 |
|  | Yes | 6 (17.1) | 9 (25) | NA | NA |  |
| Eating disorder, lifetime | No | 29 (82.9) | 28 (77.8) | 0.81 | Chi^2^ = 0.057 ; df = 1 | 0 |
|  | Yes | 6 (17.1) | 8 (22.2) | NA | NA |  |
| Family history of suicidal behaviour | No | 24 (68.6) | 21 (58.3) | 0.52 | Chi^2^ = 0.421 ; df = 1 | 0 |
|  | Yes | 11 (31.4) | 15 (41.7) | NA | NA |  |
| Number of suicide attempts |  | 0 (0) | 1.361 (0.639) | <0.0001 | F = 158.578 ; df = 1 ; dfres = 69 | 0 |
| History of lethal suicide attempt | No | 0 (NaN) | 33 (91.7) | 1 | Chi^2^ = NA ; df = NA | 0 |
|  | Yes | 0 (NaN) | 3 (8.3) | NA | NA |  |
| History of severe suicide attempt | No | 0 (NaN) | 29 (80.6) | 1 | Chi^2^ = NA ; df = NA | 0 |
|  | Yes | 0 (NaN) | 7 (19.4) | NA | NA |  |
| BDI total score |  | 17.429 (7.942) | 20.556 (6.097) | 0.07 | F = 3.475 ; df = 1 ; dfres = 69 | 0 |
| Current suicidal ideation (BDI) | No | 30 (85.7) | 28 (77.8) | 0.58 | Chi^2^ = 0.311 ; df = 1 |  |
|  | Yes | 5 (14.3) | 8 (22.2) | NA | NA |  |
| IFN-$\gamma$ | Undetected | 24 (68.6) | 21 (58.3) | 0.52 | Chi^2^ = 0.421 ; df = 1 | 0 |
|  | Detected | 11 (31.4) | 15 (41.7) | NA | NA |  |
| IL-1$\beta$ | Undetected | 17 (48.6) | 9 (25) | 0.07 | Chi^2^ = 3.293 ; df = 1 | 0 |
|  | Detected | 18 (51.4) | 27 (75) | NA | NA |  |
| IL-2 | Undetected | 25 (71.4) | 22 (61.1) | 0.5 | Chi^2^ = 0.446 ; df = 1 | 0 |
|  | Detected | 10 (28.6) | 14 (38.9) | NA | NA |  |
| IL-6 tertile | Ter 1 | 11 (31.4) | 9 (25) | 0.82 | Chi^2^ = 0.402 ; df = 2 | 0 |
|  | Ter 2 | 14 (40) | 15 (41.7) | NA | NA |  |
|  | Ter 3 | 10 (28.6) | 12 (33.3) | NA | NA |  |
| TNF-$\alpha$ tertile | Ter 1 | 14 (40) | 7 (19.4) | 0.042 | Chi^2^ = 6.333 ; df = 2 | 0 |
|  | Ter 2 | 13 (37.1) | 11 (30.6) | NA | NA |  |
|  | Ter 3 | 8 (22.9) | 18 (50) | NA | NA |  |
| PDGF-AB tertile | Ter 1 | 20 (57.1) | 11 (30.6) | 0.05 | Chi^2^ = 5.907 ; df = 2 | 0 |
|  | Ter 2 | 8 (22.9) | 17 (47.2) | NA | NA |  |
|  | Ter 3 | 7 (20) | 8 (22.2) | NA | NA |  |
| RANTES tertile | Ter 1 | 16 (45.7) | 14 (38.9) | 0.67 | Chi^2^ = 0.812 ; df = 2 | 0 |
|  | Ter 2 | 14 (40) | 14 (38.9) | NA | NA |  |
|  | Ter 3 | 5 (14.3) | 8 (22.2) | NA | NA |  |
| Uteroglobin 3c tertile | Ter 1 | 13 (37.1) | 15 (41.7) | 0.17 | Chi^2^ = 3.563 ; df = 2 | 0 |
|  | Ter 2 | 12 (34.3) | 17 (47.2) | NA | NA |  |
|  | Ter 3 | 10 (28.6) | 4 (11.1) | NA | NA |  |
| IL-4 log (imp) |  | 1.843 (1.416) | 2.062 (1.552) | 0.54 | F = 0.386 ; df = 1 ; dfres = 69 | 0 |
| Annexin log |  | -0.274 (1.701) | -0.354 (1.746) | 0.85 | F = 0.038 ; df = 1 ; dfres = 69 | 0 |
| PDGF-BB log (imp) |  | 4.675 (1.473) | 4.922 (1.051) | 0.42 | F = 0.665 ; df = 1 ; dfres = 69 | 0 |
| CRP log |  | 0.037 (0.997) | 0.07 (0.906) | 0.88 | F = 0.021 ; df = 1 ; dfres = 69 | 0 |
| TSP-2 log |  | 10.095 (0.3) | 10.1 (0.235) | 0.94 | F = 0.006 ; df = 1 ; dfres = 69 | 0 |
| MCP-1 log |  | 5.421 (0.484) | 5.501 (0.421) | 0.46 | F = 0.556 ; df = 1 ; dfres = 69 | 0 |
| Serotonin log |  | 2.369 (0.345) | 2.484 (0.519) | 0.28 | F = 1.197 ; df = 1 ; dfres = 69 | 0 |
| TGF-1$\beta$ log |  | 4.677 (0.476) | 4.723 (0.402) | 0.67 | F = 0.186 ; df = 1 ; dfres = 69 | 0 |
| TSP-1 log |  | 12.085 (0.718) | 12.052 (0.655) | 0.84 | F = 0.041 ; df = 1 ; dfres = 69 | 0 |
| Centrin 2 log |  | -0.609 (0.687) | -0.795 (0.664) | 0.25 | F = 1.353 ; df = 1 ; dfres = 69 | 0 |
| GFAP tercile | Ter 1 | 8 (22.9) | 12 (34.3) | 0.32 | Chi^2^ = 2.268 ; df = 2 | 0 |
|  | Ter 2 | 17 (48.6) | 11 (31.4) | NA | NA |  |
|  | Ter 3 | 10 (28.6) | 12 (34.3) | NA | NA |  |
| GFAP log |  | 4.35 (0.34) | 4.258 (0.494) | 0.37 | F = 0.816 ; df = 1 ; dfres = 69 | 0 |
| NFL tercile | Ter 1 | 10 (28.6) | 9 (25.7) | 0.59 | Chi^2^ = 1.053 ; df = 2 | 0 |
|  | Ter 2 | 10 (28.6) | 14 (40) | NA | NA |  |
|  | Ter 3 | 15 (42.9) | 12 (34.3) | NA | NA |  |
| NFL log |  | 2.155 (0.504) | 2.155 (0.57) | 1 | F = 0 ; df = 1 ; dfres = 69 | 0 |
| CD3% in single cells |  | 20.002 (5.001) | 20.46 (6.629) | 0.75 | F = 0.102 ; df = 1 ; dfres = 65 | 5 |
| CD3% in single cells (imp) |  | 19.89 (4.971) | 20.411 (6.387) | 0.7 | F = 0.147 ; df = 1 ; dfres = 69 | .. |
| CD3% in CD45 |  | 69.913 (7.342) | 68.893 (9.943) | 0.63 | F = 0.232 ; df = 1 ; dfres = 66 | 3 |
| CD3% in CD45 (imp) |  | 69.399 (7.847) | 69.012 (9.705) | 0.85 | F = 0.034 ; df = 1 ; dfres = 69 | .. |
| CD14% in CD45 |  | 7.988 (4.833) | 7.785 (4.748) | 0.86 | F = 0.031 ; df = 1 ; dfres = 66 | 3 |
| CD14% in CD45 (imp) |  | 8.104 (4.81) | 7.737 (4.63) | 0.74 | F = 0.107 ; df = 1 ; dfres = 69 | .. |
| CD4% in CD3 |  | 61.543 (9.125) | 57.077 (10.516) | 0.07 | F = 3.454 ; df = 1 ; dfres = 65 | 4 |
| CD4% in CD3 (imp) |  | 61.425 (9.016) | 57.186 (10.139) | 0.07 | F = 3.459 ; df = 1 ; dfres = 69 | .. |
| CD8% in CD3 |  | 29.516 (7.991) | 32.614 (7.897) | 0.12 | F = 2.545 ; df = 1 ; dfres = 65 | 4 |
| CD8% in CD3 (imp) |  | 29.693 (7.941) | 32.707 (7.614) | 0.11 | F = 2.665 ; df = 1 ; dfres = 69 | .. |
| CD4/CD8 ratio |  | 2.35 (1.225) | 1.895 (0.679) | 0.07 | F = 3.497 ; df = 1 ; dfres = 65 | 4 |
| Nucleated cell count |  | 6.862 (1.56) | 7.292 (2.119) | 0.35 | F = 0.89 ; df = 1 ; dfres = 65 | 4 |
| Platelet count |  | 248.118 (55.868) | 253.657 (71.408) | 0.72 | F = 0.128 ; df = 1 ; dfres = 67 | 2 |
| Neutrophil count |  | 4.163 (1.242) | 4.689 (1.974) | 0.2 | F = 1.697 ; df = 1 ; dfres = 65 | 4 |
| Eosinophil count |  | 0.181 (0.115) | 0.187 (0.136) | 0.84 | F = 0.04 ; df = 1 ; dfres = 65 | 4 |
| Eosinophil count (imp) |  | 0.186 (0.114) | 0.181 (0.137) | 0.85 | F = 0.038 ; df = 1 ; dfres = 69 | .. |
| Basophil count |  | 0.052 (0.024) | 0.053 (0.019) | 0.79 | F = 0.073 ; df = 1 ; dfres = 65 | 4 |
| Basophil count (imp) |  | 0.052 (0.023) | 0.052 (0.019) | 0.98 | F = 0.001 ; df = 1 ; dfres = 69 | .. |
| Lymphocyte cunt |  | 1.987 (0.546) | 1.861 (0.498) | 0.33 | F = 0.967 ; df = 1 ; dfres = 65 | 4 |
| Monocyte count |  | 0.481 (0.132) | 0.502 (0.171) | 0.57 | F = 0.321 ; df = 1 ; dfres = 65 | 4 |
| Blood NLR |  | 2.22 (0.813) | 2.714 (1.353) | 0.08 | F = 3.249 ; df = 1 ; dfres = 65 | 4 |
| Blood NLR (imp) |  | 2.195 (0.811) | 2.707 (1.314) | 0.05 | F = 3.876 ; df = 1 ; dfres = 69 | .. |
| Blood PLR |  | 131.293 (40.625) | 143.38 (39.58) | 0.22 | F = 1.522 ; df = 1 ; dfres = 65 | 4 |
| Blood PLR (imp) |  | 131.337 (39.558) | 141.371 (39.358) | 0.29 | F = 1.148 ; df = 1 ; dfres = 69 | .. |
| Blood MLR |  | 0.25 (0.068) | 0.293 (0.154) | 0.15 | F = 2.149 ; df = 1 ; dfres = 65 | 4 |
| Blood MLR (imp) |  | 0.248 (0.07) | 0.293 (0.15) | 0.11 | F = 2.665 ; df = 1 ; dfres = 69 | .. |
| MFA Dim 1 |  | -0.667 (1.062) | -0.105 (1.335) | 0.05 | F = 3.849 ; df = 1 ; dfres = 69 | .. |
| MFA Dim 2 |  | 0.204 (1.14) | 0.344 (1.407) | 0.65 | F = 0.212 ; df = 1 ; dfres = 69 | .. |
| MFA Dim 3 |  | 0.125 (0.941) | 0.051 (1.307) | 0.78 | F = 0.076 ; df = 1 ; dfres = 69 | .. |
| Treatment |  |  |  |  |  |  |
| Anxiolytic or hypnotic | No | 13 (37.1) | 4 (11.1) | 0.02 | Chi^2^ = 5.252 ; df = 1 | 0 |
|  | Yes | 22 (62.9) | 32 (88.9) | NA | NA |  |
| Antidepressant | No | 6 (17.1) | 10 (27.8) | 0.43 | Chi^2^ = 0.621 ; df = 1 | 0 |
|  | Yes | 29 (82.9) | 26 (72.2) | NA | NA |  |
| Antipsychotic | No | 21 (60) | 14 (38.9) | 0.12 | Chi^2^ = 2.376 ; df = 1 | 0 |
|  | Yes | 14 (40) | 22 (61.1) | NA | NA |  |
| Mood stabilizer | No | 21 (60) | 14 (38.9) | 0.12 | Chi^2^ = 2.376 ; df = 1 | 0 |
|  | Yes | 14 (40) | 22 (61.1) | NA | NA |  |

Non-adjusted pairwise comparisons. P-values are two-sided.

MDE, major depressive episode; SA, suicide attempters.
