# Supplementary material for: Integrating cellular and soluble immune signatures of major depression with and without recent suicide attempts
Source: Transl Psychiatry. 2025 Oct 6;15:377. doi: 10.1038/s41398-025-03601-2 (PMC12501231; doi:10.1038/s41398-025-03601-2)
Supplement: Supplementary file 7 — Supplemental Table S6 [file 41398_2025_3601_MOESM7_ESM.docx]

Supplemental Table S6. Multivariate associations between study groups and MFA dimensions.

|  | HC vs MDE | | HC vs SA | | MDE vs SA | |
| --- | --- | --- | --- | --- | --- | --- |
| Dimension | OR (95% CI) | LRT p-value | OR (95% CI) | LRT p-value | OR (95% CI) | LRT p-value |
| First | 0.206 (0.08 to 0.433) | <0.0001 | 0.303 (0.14 to 0.585) | 0.0002 | 1.652 (0.965 to 2.953) | 0.07 |
| Second | 2.689 (1.333 to 6.249) | 0.005 | 3.305 (1.693 to 7.716) | 0.0002 | 0.993 (0.586 to 1.701) | 0.98 |
| Third | 2.519 (1.225 to 6.055) | 0.01 | 1.644 (0.881 to 3.286) | 0.12 | 0.894 (0.512 to 1.527) | 0.68 |

Abbreviations: LRT, likelihood ratio test; MDE, major depressive disorder, without suicide attempt history; HC, healthy controls; OR, Odds Ratio; SA, suicide attempters.

Multivariate associations are estimated from logistic regression, adjusted for sex and age.
