# Supplementary figures and images for: Integrating cellular and soluble immune signatures of major depression with and without recent suicide attempts
Source: Transl Psychiatry. 2025 Oct 6;15:377. doi: 10.1038/s41398-025-03601-2 (PMC12501231; doi:10.1038/s41398-025-03601-2)

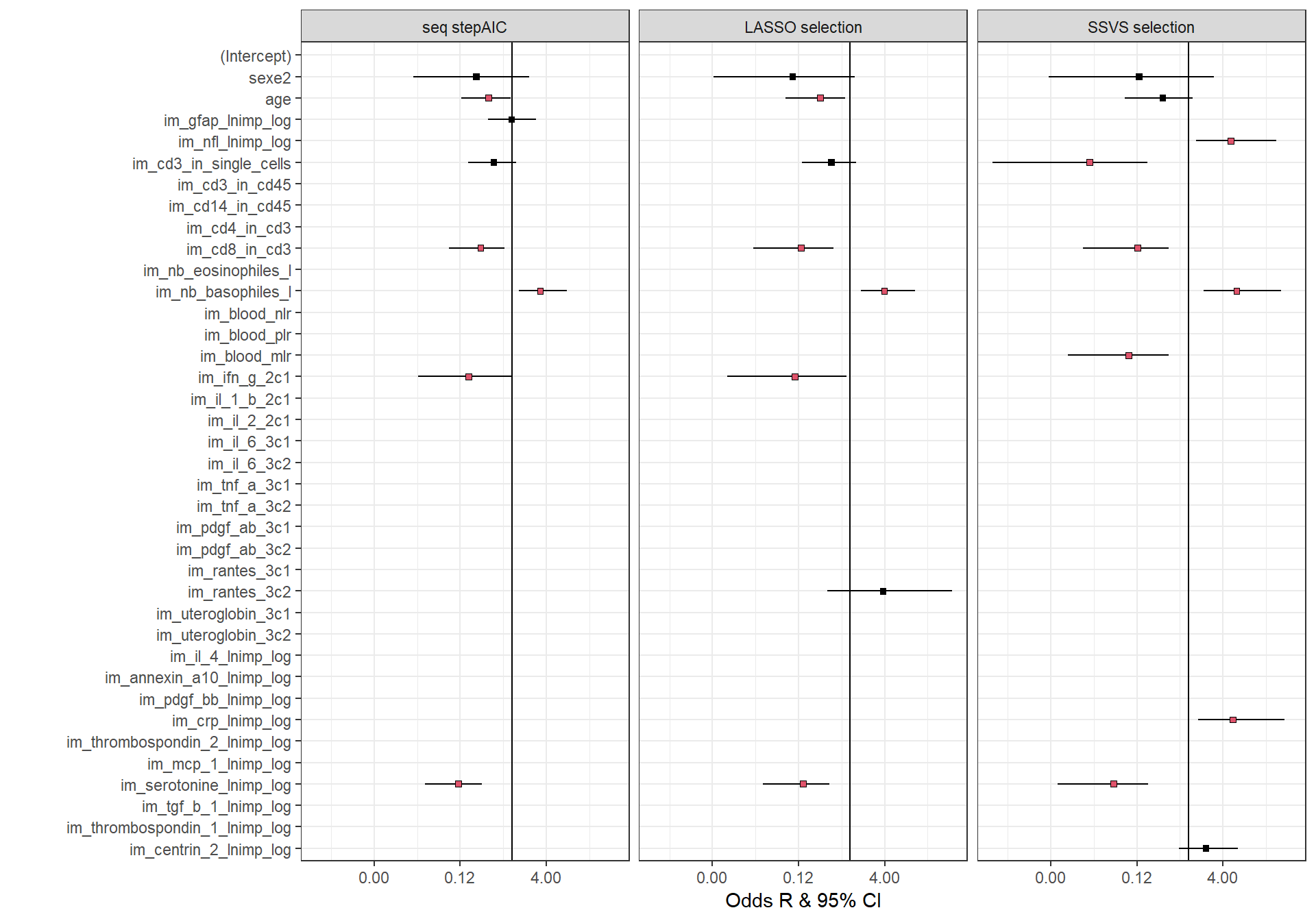

Supplement: Supplementary file 8 — Supplemental Figure S1 [file 41398_2025_3601_MOESM8_ESM.png]

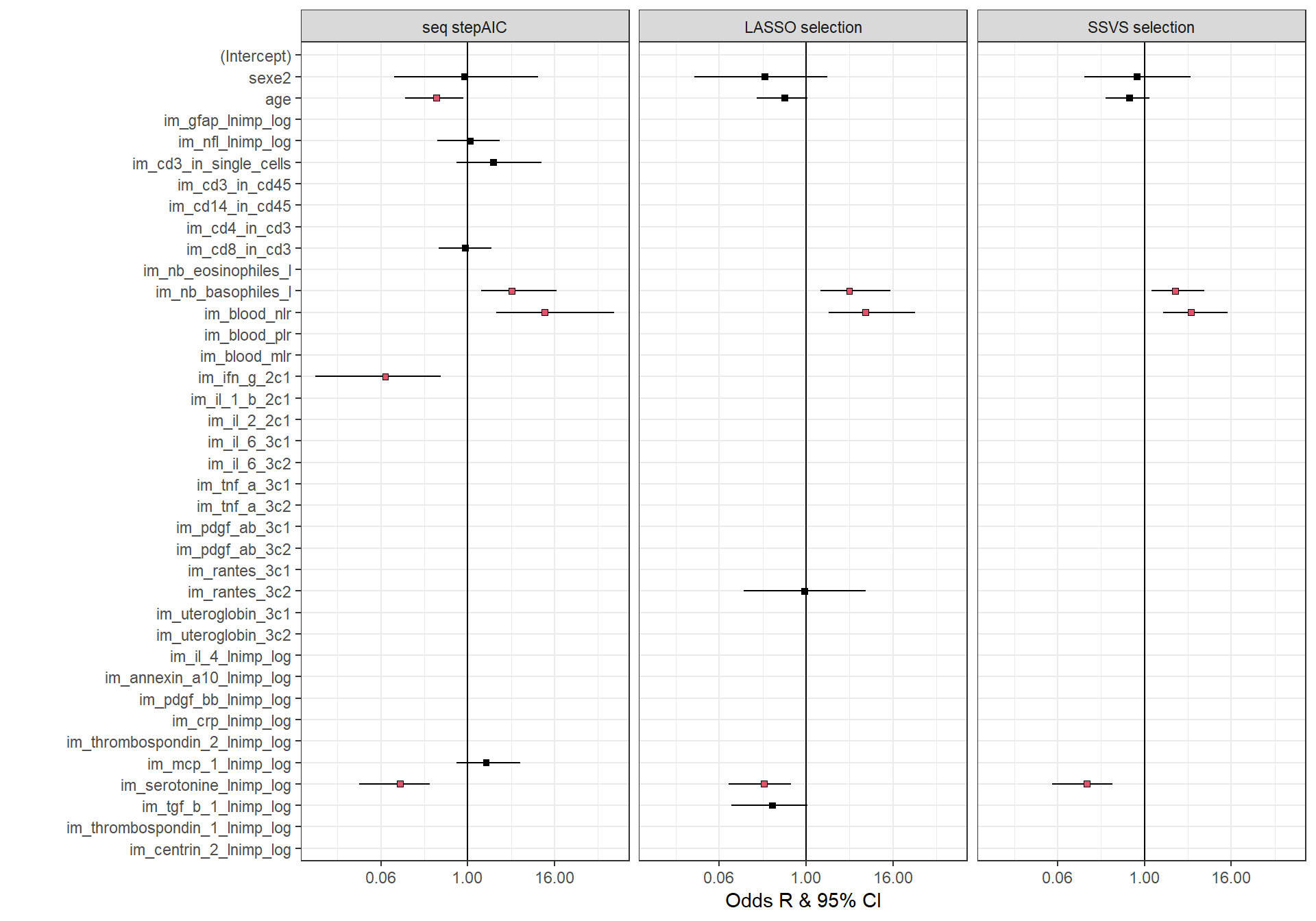

Supplement: Supplementary file 9 — Supplemental Figure S2 [file 41398_2025_3601_MOESM9_ESM.png]

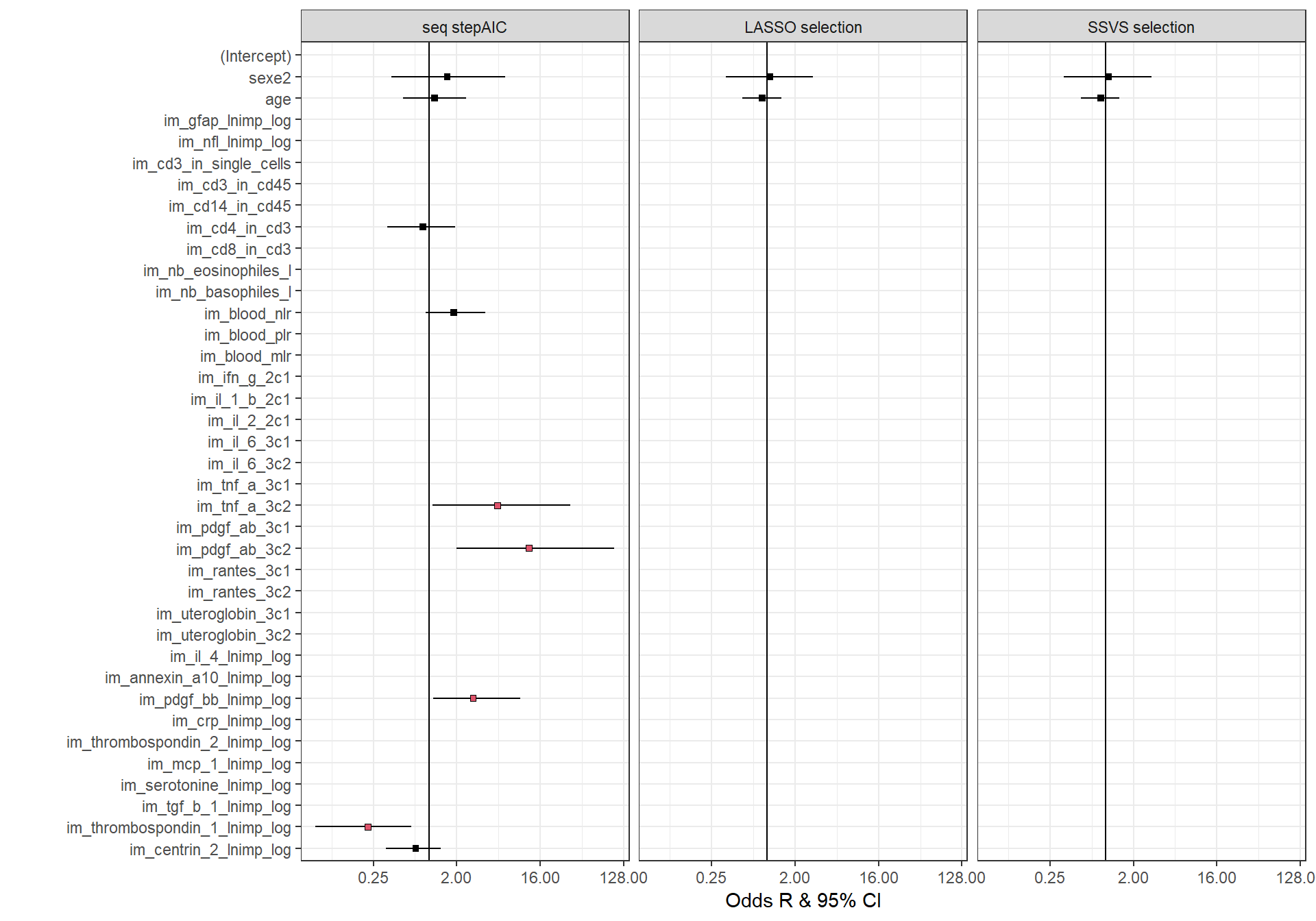

Supplement: Supplementary file 10 — Supplemental Figure S3 [file 41398_2025_3601_MOESM10_ESM.png]
