# Supplementary material for: Integrating cellular and soluble immune signatures of major depression with and without recent suicide attempts
Source: Transl Psychiatry. 2025 Oct 6;15:377. doi: 10.1038/s41398-025-03601-2 (PMC12501231; doi:10.1038/s41398-025-03601-2)

Supplemental Figure 5. Characterization of the three meaningful Multiple Factor analysis dimensions in a subsample of patients with major depressive disorder.


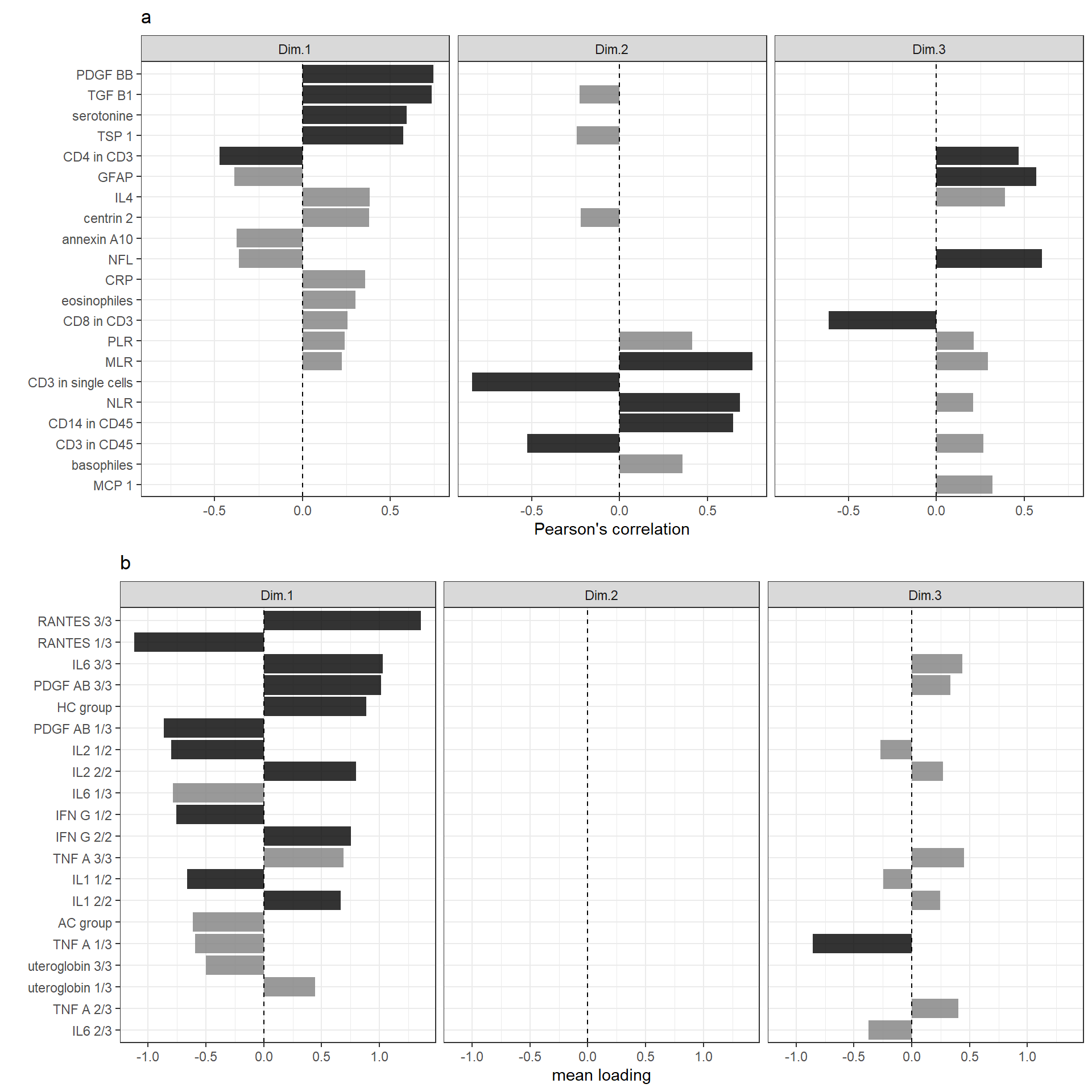

Supplement: Supplementary file 12 — Supplemental Figure S5 [file 41398_2025_3601_MOESM12_ESM.docx]
