# Supplementary material for: Integrating cellular and soluble immune signatures of major depression with and without recent suicide attempts
Source: Transl Psychiatry. 2025 Oct 6;15:377. doi: 10.1038/s41398-025-03601-2 (PMC12501231; doi:10.1038/s41398-025-03601-2)
Supplement: Supplementary file 13 — Supplemental Figure S6 [file 41398_2025_3601_MOESM13_ESM.docx]

Supplemental Figure 6. Odds ratios and 95% confidence intervals from multivariate models following the SSVS selection procedure for the three group comparisons. Sex and age were included in the models as potentially confounding factors.


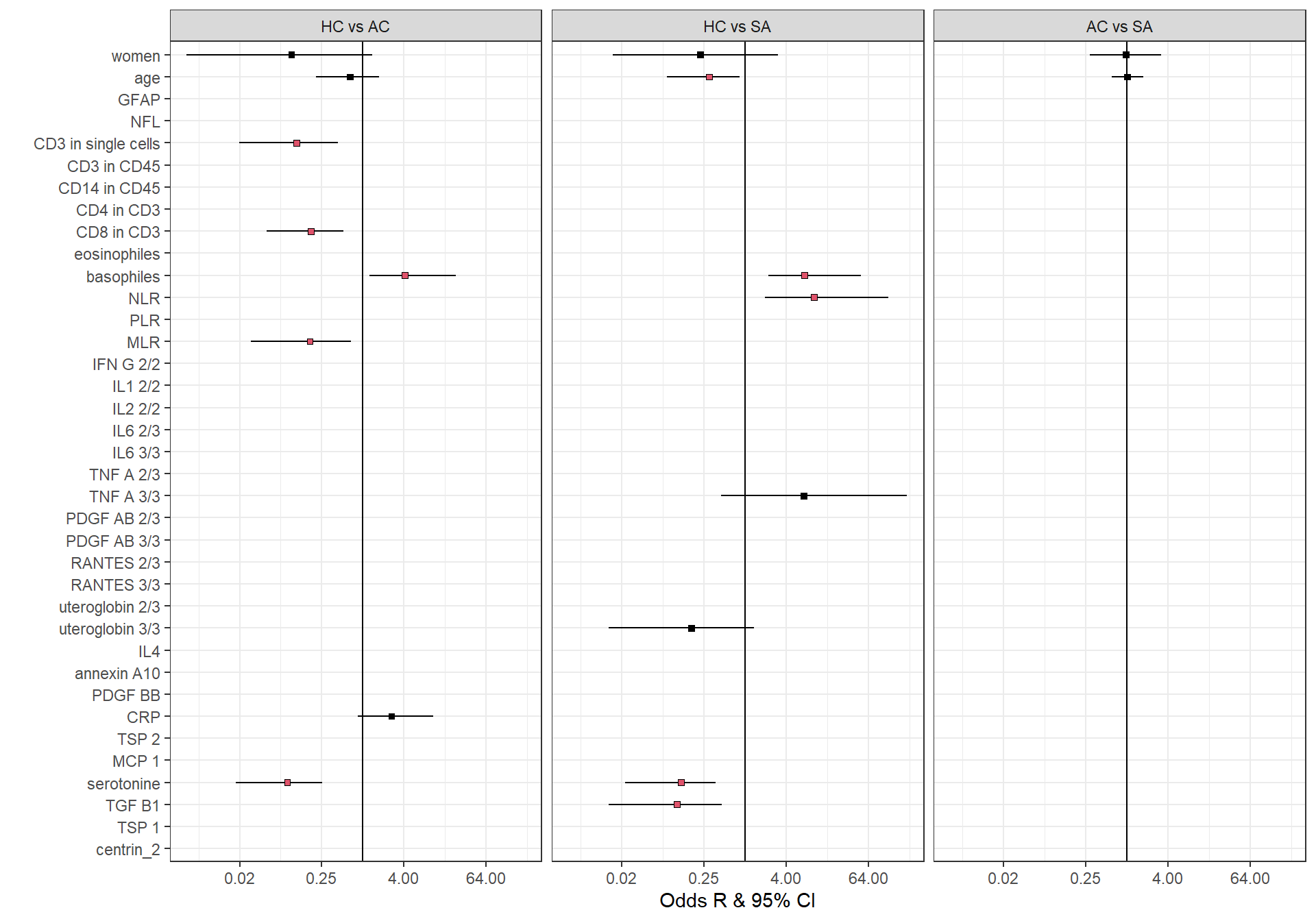


Odds ratios and confidence intervals for the SSVS method. Red points represent significant effects of variables, while black points represent non-significant variables retained in the models. Sex and age were included as forced variables. The SSVS model was selected as the representative model.
