# Supplementary material for: Integrating cellular and soluble immune signatures of major depression with and without recent suicide attempts
Source: Transl Psychiatry. 2025 Oct 6;15:377. doi: 10.1038/s41398-025-03601-2 (PMC12501231; doi:10.1038/s41398-025-03601-2)
Supplement: Supplementary file 14 — Supplemental table S7 [file 41398_2025_3601_MOESM14_ESM.docx]

Supplemental Table S7. Multivariate associations between study groups and MFA dimensions in a subgroup of patients with major depressive disorder.

|  | HC vs MDE | | HC vs SA | | MDE vs SA | |
| --- | --- | --- | --- | --- | --- | --- |
| Dimension | OR (95% CI) | LRT p-value | OR (95% CI) | LRT p-value | OR (95% CI) | LRT p-value |
| First | 0.176 (0.062 to 0.393) | <0.0001 | 0.234 (0.101 to 0.471) | <0.0001 | 1.36 (0.773 to 2.467) | 0.27 |
| Second | 2.077 (1.057 to 4.559) | 0.003 | 2.194 (1.173 to 4.79) | 0.01 | 1.016 (0.587 to 1.783) | 0.95 |
| Third | 2.035 (0.997 to 4.703) | 0.05 | 1.476 (0.765 to 3.064) | 0.25 | 0.985 (0.55 to 1.756) | 0.96 |

Abbreviations: LRT, likelihood ratio test; MDE, major depressive disorder, without suicide attempt history; HC, healthy controls; OR, Odds Ratio; SA, suicide attempters.

Multivariate associations are estimated from logistic regression, adjusted for sex and age.
